# Engineered Factor VIII: A Comprehensive Review of Molecular Biology, Design Strategies, and AAV-Mediated Gene Therapy

**DOI:** 10.3390/ijms27219928

**Published:** 2026-11-05

**Authors:** Albina Khairetdinova, Valentin Artemyev, Ekaterina A. Astakhova, Syune Ambaryan, Matvey Pilagov, Anna Maznina, Victoria S. Lobanova, Sofya G. Feoktistova, Olga N. Mityaeva, Pavel Yu. Volchkov

**Affiliations:** 1 Federal Research Center for Innovator and Emerging Biomedical and Pharmaceutical Technologies, Moscow 125315, Russia; astakhova_ea@academpharm.ru (E.A.A.); ambarjan.sh@genlab.llc (S.A.); pilagov.mn@genlab.llc (M.P.); sayfutdinovas@gmail.com (S.G.F.); mityaeva_on@academpharm.ru (O.N.M.); 2 Moscow Center for Advanced Studies, Kulakova str. 20, Moscow 123592, Russia; maznina@genlab.llc; 3 Morozov’s Moscow City Children Clinical Hospital, Beregovoy Proezd 5A/1, Moscow 121087, Russia; kakaulinavs@gmail.com; 4 Moscow Clinical Scientific Center N.A. A.S. Loginov, Moscow 111123, Russia; volchkov_py@academpharm.ru

**Keywords:** hemophilia A, gene therapy, adeno-associated virus, coagulation factor VIII, protein engineering, expression enhancement, hemophilia A clinical trials, liver-directed therapy

## Abstract

Coagulation factor VIII (FVIII) is a key therapeutic protein for hemophilia A, an inherited bleeding disorder caused by FVIII deficiency or dysfunction. The development and production of FVIII-based therapeutics remains challenging due to the intrinsic complexity of FVIII biosynthesis, folding, secretion, and functional stability. Advances in the understanding of the FVIII life cycle, as well as studies of its natural homologs, have created opportunities to develop improved FVIII variants. In parallel, therapeutic strategies to restore FVIII activity have evolved from replacement therapy with exogenous FVIII to gene-based delivery of a functional *F8* copy. Among these approaches, AAV-mediated gene therapy has emerged as a clinically viable platform with a generally favorable safety profile, prompting the development of multiple therapeutic candidates for hemophilia A. This review summarizes established and current knowledge of FVIII structure, biosynthesis, and immunology in the context of developing effective and clinically applicable AAV-mediated therapies for hemophilia A. Particular attention is given to engineered FVIII variants and the latest clinical findings in AAV gene therapy, as well as to long-term efficacy and safety.

## 1. Introduction

Hemophilia is the most common inherited X-linked disorder of hemostasis, associated with a deficiency of factor VIII (FVIII) or factor IX in hemophilia A (HA) or in hemophilia B (HB), respectively. HA has a much higher prevalence (1 in 5000 in males) than HB and accounts for 85% of all cases of hemophilia, with a prevalence of 1 in 5000 males [1]. Various mutations in the *F8* gene determine the different disease severities, and depending on FVIII (FVIII:C) activity, HA is classified as severe (FVIII:C < 1%), moderate (FVIII:C = 1–5%), or mild (FVIII:C = 5–40%). According to the European Haemophilia Safety Surveillance (EUHASS) registry, the prevalence of HA forms is as follows: 40.5% severe, 10% moderate, and 50% mild [2].

The mild form of hemophilia is characterized by rare spontaneous bleeding or excessive bleeding following trauma or surgery. However, as FVIII activity decreases, the frequency of spontaneous bleeding (ABR, annualized bleeding rates) increases significantly, averaging up to 24 bleeds per year [3]. The bleeding severity and the consequences are location-dependent. Joint bleeding (70–80%), the most common type, leads to hemarthrosis and the development of hemophilic arthropathy. While rare (<5%), bleeding into the nervous system is the leading cause of death in individuals with severe HA [1]. Importantly, spontaneous bleeding observed in severe hemophilia patients is more common in HA than in HB, despite equal procoagulant activity. In fact, patients with HB undergo invasive orthopedic procedures to restore joint function 30% less frequently [4]. Meanwhile, joint diseases are among the most disabling and costly complications of severe hemophilia [3].

**Figure 1:**
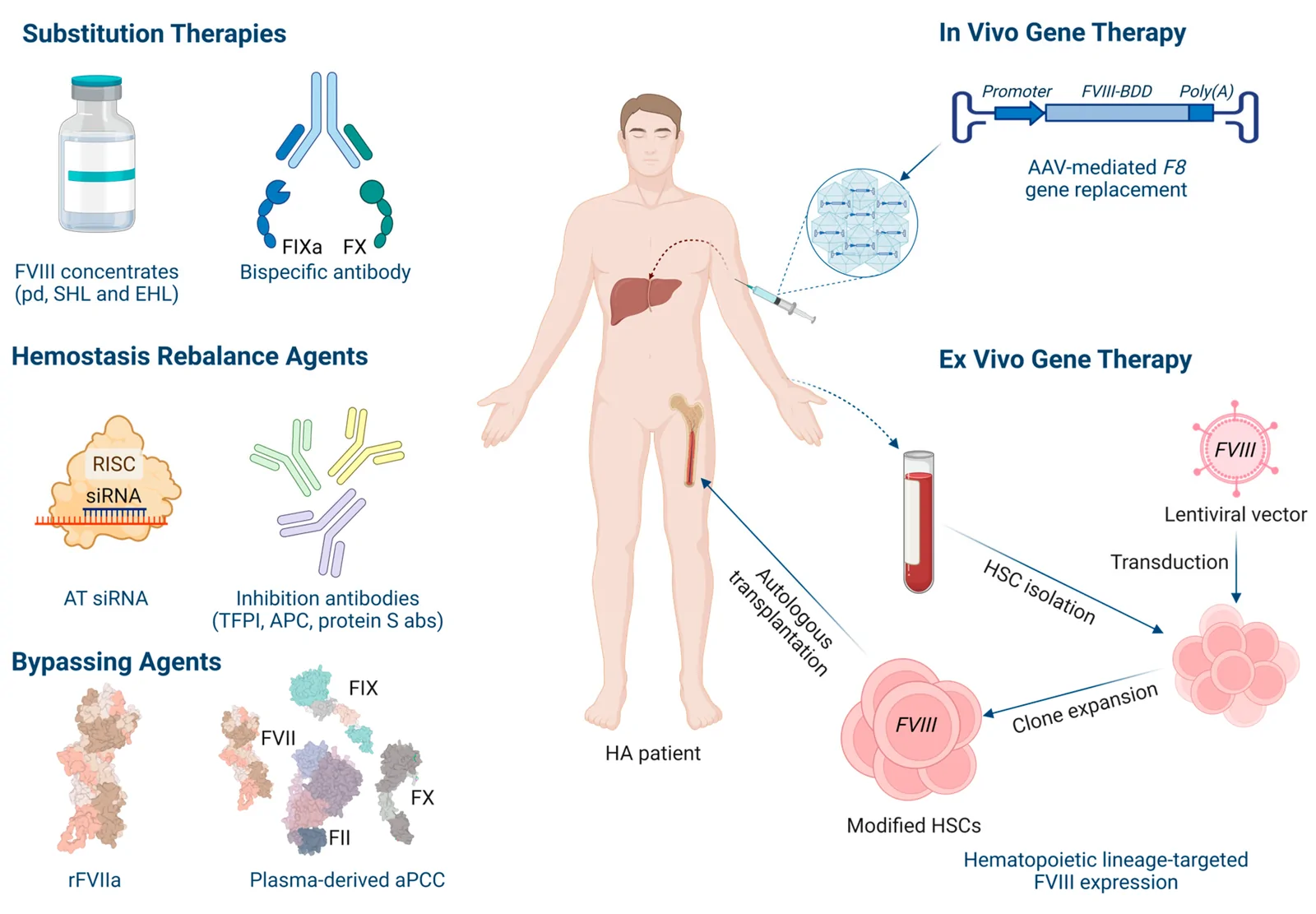
Hemophilia A treatment approaches.

Various approaches can be used to manage HA, depending on disease severity. For example, desmopressin acetate (a synthetic analog of vasopressin) can be used in cases of mild HA to increase plasma concentrations of FVIII and von Willebrand factor (vWF). Up to half of severe HA cases are attributed to inversions of introns 1 and 22 of the *F8* gene, which prevent the expression of the protein even in a dysfunctional form; therefore, HA treatment requires medications that restore FVIII function [1]. The current standard of care for HA is the prophylactic or on-demand administration of plasma-derived (pd) or recombinant FVIII with a standard half-life (SHL) or enhanced half-life (EHL) (Figure 1) [5]. EHL-rFVIII has been reported to be more effective for long-term prophylaxis in previously treated patients, since it is associated with an 88% decreased risk of inhibitor development compared to therapy using SHL-rFVIII and pdFVIII [6]. A plausible explanation may be steric masking of immunogenic FVIII epitopes and/or less frequent injections of concentrates [7].

New non-factor therapies have become available for the treatment of HA in recent years. Emicizumab became the first non-factor therapy for the prophylaxis of patients with HA, although several similar compounds are currently in clinical trials [8,9]. The compound is a bispecific antibody that substitutes for the function of FVIII by ensuring the interaction between FIX and FX [10,11]. An alternative way to treat hemophilia is to rebalance hemostasis by altering the normal coagulation cascade through the inhibition of natural anticoagulants. These medicines include Fitusiran, a small interfering RNA that inhibits antithrombin (AT) expression in the liver, and monoclonal antibodies against tissue factor pathway inhibitor (TFPI), activated protein C (APC), and protein S [12]. An obvious advantage of the hemostasis rebalancing strategy is its suitability for both types of hemophilia and its independence from the presence of neutralizing antibodies (nAbs) [13]. In contrast, rebalancing agents cannot completely eliminate bleeding episodes, and their use is limited by the risks of uncontrolled thrombin generation [12,14,15,16]. It has been found to be helpful to determine FVIII or FIX equivalence before starting treatment with these drugs. However, this poses a challenge because rebalancing agents interfere with many standard coagulation assays [12]. Due to the distinct action mechanisms, only the actual bleeding rate (ABR), not FVIII activity, can reliably serve as a therapeutic measure of a drug’s efficacy when comparing different treatment options [3].

Bypassing agents represent a distinct class of medicines used in cases of acute bleeding in patients with nAbs that prevent the use of FVIII concentrates. In contrast to rebalancing agents, which reduce the activity of anticoagulants and thereby restore hemostatic balance, bypassing agents attempt to enhance procoagulant pathways by bypassing FVIII. These drugs include recombinant activated coagulation factor VII (rVIIa) and plasma-derived aPCC [13].

Gene therapy has emerged as a novel class of treatments for hereditary diseases. HA is a common model for the development of gene therapies, designed to deliver a functional copy of the *F8* gene, based on the monogenic origin of the disease and its broad therapeutic window, with an increase in activity to just 5%, significantly improving clinical outcomes [17]. Two therapies have been approved by the FDA (Hemgenix, Beqvez) and one in China (BBM-H901) for HB, whereas only one therapy has been approved for HA (Roctavian), which was recently withdrawn due to commercial underperformance [15]. This is attributed to a number of properties of factor VIII, including the great length of its coding sequence, low expression efficacy, and a high frequency of nAbs formation in patients with HA, which significantly affect the efficacy and safety of gene therapies. This explains the strong interest in studying the properties of FVIII and designing new synthetic variants that can be applied in emerging gene therapies and help a greater number of individuals with HA.

## 2. Coagulation Factor FVIII: From Synthesis to Inactivation

### 2.1. F8 Gene and mRNA

**Figure 2:**
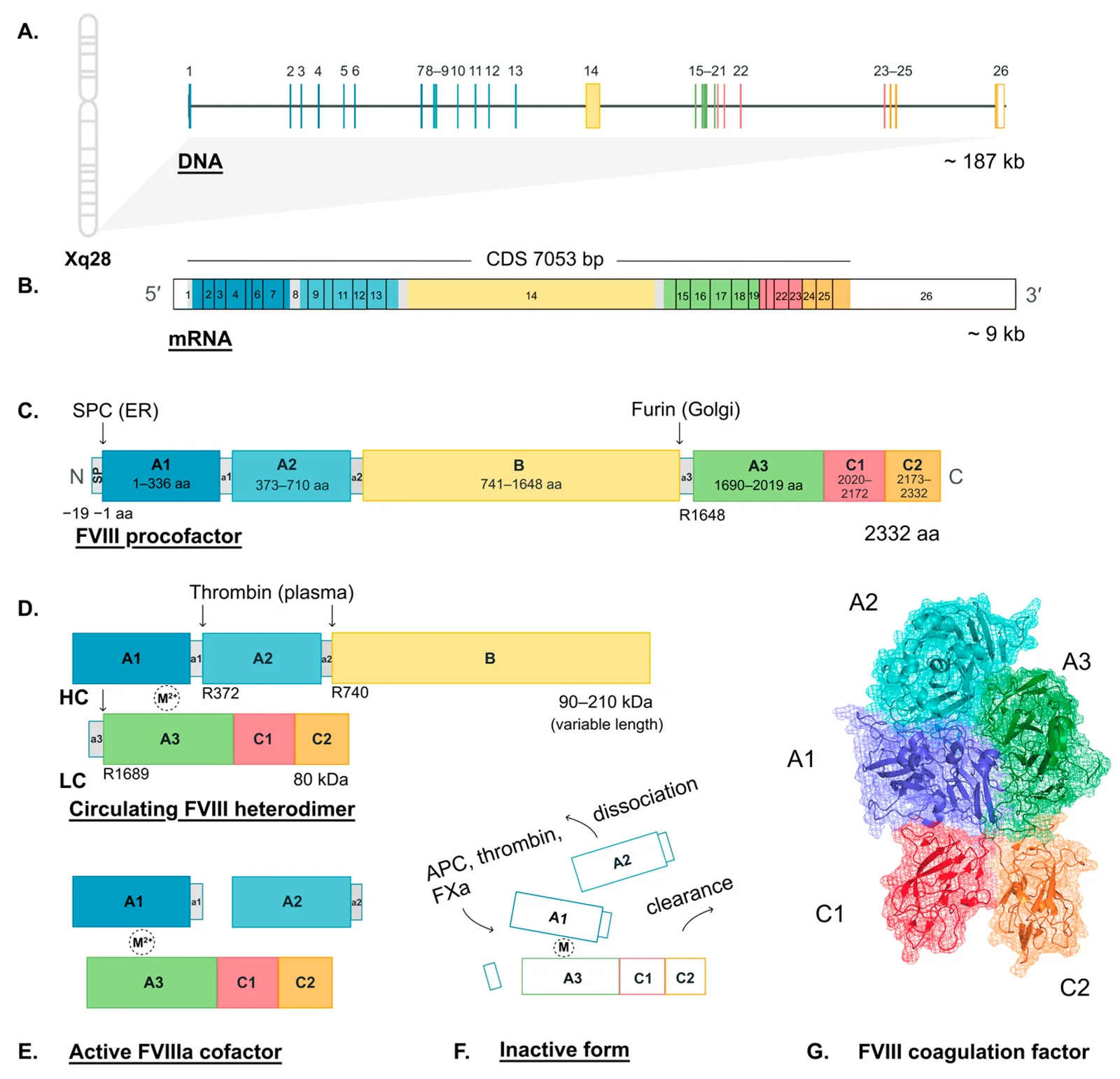
Factor VIII gene and protein structures. (**A**)—The human *F8* gene on chromosome Xq28. (**B**)—The *F8* mRNA transcript with the FVIII coding sequence distributed across 26 exons. The transcript is approximately 9 kb in length and contains a 7053 bp coding sequence distributed across 26 exons. (**C**)—Domain organization and intracellular processing of the FVIII procofactor. Mature full-length FVIII after SP cleavage comprises 2332 a.a. (**D**)—FVIII circulates as a heterodimer consisting of the heavy chain (HC) and the light chain (LC). (**E**)—Thrombin activation releases the B domain and generates the heterotrimeric FVIIIa. (**F**)—The A2 subunit can dissociate spontaneously from the FVIIIa complex, resulting in loss of cofactor activity. APC, thrombin, or FXa promotes FVIIIa inactivation and subsequent clearance of FVIII-derived fragments. (**G**)—The three-dimensional structure of FVIII. The B domain is not represented because it is removed during activation and is structurally flexible in the procofactor.

The F8 gene is located in the distal arm of the X chromosome (Xq28) [18,19]. In the GRCh38.p14 assembly, F8 spans positions 154,835,792–155,022,723 on the complementary strand and comprises approximately 186 kb, including 26 exons (Figure 2) (PubMed, Gene ID: 2157).

*F8* has two known promoter elements. The first promoter element drives transcription of the mature canonical mRNA of approximately 9 kb (NM_000132.4). This transcript contains a 7053 bp coding sequence that encodes the full-length 2351 amino acid (a.a.) FVIII protein, with the 5′- and the long 3′-UTR accounting for 171 bp and 1.8 kb, respectively [18,19,20]. The second promoter is located within intron 22 of the *F8* gene and drives expression of an approximately 2.6 kb truncated transcript (NM_019863.3), with an alternative first exon that is not present in the canonical transcript. The resulting predicted sequence is small, 216 a.a. long, and expected to carry 8 unique N-terminal residues plus residues 2144–2351 of the FVIII phospholipid-binding domains, although it has not yet been detected in any proteomic study to date [19]. Several additional alternative truncated transcripts of undetermined functional significance have also been reported: two short pulmonary-endothelium isoforms that use alternative 5′ exons located within intron 22, as well as two variants that initiate from exon U1, positioned 4.3 kb upstream of the canonical first exon [21]. Exon-skipping variants have, likewise, been found. The exon-22-skipped (ΔE22) transcript was identified at low abundance in leukocytes from both healthy individuals and HA-affected patients. In silico modeling suggests at least a partial loss of the β-sheet in the FVIII C1 domain, though no study has yet established whether FVIII-ΔE22 is functionally relevant [22].

Low-level expression of FVIII mRNA is observed across various tissues and organs but is most represented in the liver, lungs, heart, lymph nodes, and adipose tissue and is predominantly localized to endothelial cells and adipocytes [19,23,24]. The main source of functional protein is the liver sinusoidal endothelial cell (LSEC) population in both the fetal and adult liver [25,26]. These cells secrete sufficient protein to maintain normal plasma FVIII levels [21]. However, *F8* transcription remains relatively low, even in LSECs, and is substantially lower than that of genes encoding many other coagulation factors.

Early *F8* mRNA expression has been detected in the liver and heart at 6–10 weeks of gestation, reaching up to 50% of adult levels (the reported transcript was approximately 8.1 kb in length) [27]. Consistent with the progressive maturation of the fetal liver, *F8* expression is increased at six months of gestation [28].

Cis-regulatory elements with transcription-repressive activity have been described within the *F8* coding sequence [29,30]. The most characterized *F8* repressor spans nucleotides 1546–1850 within the A2-domain coding region of the canonical *F8* transcript (NM_000132.4) and mediates an approximately tenfold reduction in reporter-gene expression [29]. In 2021, a variant designated FVIII-Padua was identified in association with a 23,420 bp tandem duplication encompassing the *F8* promoter, exon 1, and most of intron 1. This duplicated region was found to contain open-chromatin enhancer elements, consistent with the more than 2-fold rise in *F8* mRNA expression and the >250–400% FVIII:Ag (FVIII antigen level) and FVIII:C observed during the experiment [31]. Subsequent work further mapped the enhancer activity to a 400 bp fragment within intron 1 [32]. Although the FVIII-Padua variant showed only a 2.5- to 4-fold increase in plasma antigen during FVIII expression in vitro, testing this enhancer in reporter systems demonstrated a potential 10- to 45-fold transgene expression enhancement using the luciferase assay [31,32]. This discrepancy raises the possibility that FVIII biosynthesis and secretion restrict the conversion of increased *F8* transcription into FVIII protein output.

### 2.2. FVIII Intracellular Expression

#### 2.2.1. FVIII Domain Structure

FVIII is synthesized as a secreted 2351-residue glycoprotein with the domain architecture signal peptide (SP) -A1-a1-A2-a2-B-a3-A3-C1-C2, where a1–a3 are acidic linker regions. It shares similar domain organization with coagulation factor V (FV) and exhibits approximately 40% sequence homology. Following signal peptide cleavage, FVIII undergoes folding and quality control in the endoplasmic reticulum (ER), followed by glycan maturation, tyrosine sulfation, and limited proteolysis in the Golgi apparatus. It is then secreted predominantly as a noncovalent heterodimer comprising a length-variable heavy chain (HC) A1-a1-A2-a2-B and a light chain (LC) a3-A3-C1-C2. The A domains form the functional scaffold of FVIIIa, whereas the discoidin-like C1/C2 domains mediate interactions with vWF, phospholipid membranes, and the intrinsic tenase complex (Xase) [33,34]. High-resolution cryo-EM studies published in recent years have substantially refined current understanding of FVIII structural organization [35]. In plasma, vWF stabilizes FVIII and reduces its clearance. Upon thrombin-mediated activation, FVIII is released and functions as a cofactor for FIXa within the intrinsic Xase complex.

FVIII residues can be numbered either according to the Human Genome Variation Society (HGVS convention), starting from Met1, or according to the widely used legacy convention, in which numbering begins at the N-terminus of the mature protein (HGVS residue 20) and SP residues are designated −19 to −1. For consistency with the literature that historically shaped the field, legacy numbering is used throughout this review [36,37].

Current understanding of the intracellular FVIII trafficking route has been altered in light of recent studies, as this framework enables low protein productivity to be viewed as a set of potentially optimizable bottlenecks.

#### 2.2.2. FVIII B-Domain

The B-domain is the largest region of the factor VIII molecule, encoded by 908 a.a. residues and accounting for 39% of the whole protein sequence. In plasma, inactive FVIII is present as a heterogeneous population of molecules containing fully or partially cleaved C-terminal B-domain fragments, generated by proteolytic cleavage at Arg817, Arg1115, Arg1313, and Arg1648. However, complete release of the B domain during thrombin-mediated activation requires cleavage at Arg740 [20,37]. Structurally, the B domain is highly hydrophilic, with hydrophobic residues distributed relatively evenly across its sequence [38], and, like both the FVIII orthologues and the FV homolog, is heavily N-glycosylated at the asparagine residues, accounting for approximately ¾ of all FVIII glycosylation sites [39]. It also contains most of the protein’s O-glycosylation sites, which may be defined by its presumed intrinsic disordered structure and consequent enzyme availability [40]. Unlike the other FVIII domains, the B-domain a.a. sequence is poorly conserved among orthologues [20,39].

Despite containing several intracellular processing sites, numerous in vitro expression, preclinical, and clinical studies have shown that the B domain is not required for FVIII secretion or procoagulant activity [41,42,43]. The role of the free B domain in the bloodstream following its cleavage remains unclear, and there are no data on its biological or regulatory functions. Lacking a stable structure, it does not crystallize [39], and most of what is known about B-domain properties comes from heterologous expression systems [37]. The main proposed functions of the FVIII B domain are described below.

***Prevention of FVIII aggregation***. B-domain truncation increases the propensity of FVIII to form cross-β-sheet-rich aggregates in vitro, in proportion to the extent of truncation. This was demonstrated in heat-induced aggregation assays, in which aggregation became detectable above 35 °C [38]. Such a protective mechanism, whereby a natively disordered domain provides polar interactions that prevent aggregation of folding intermediates, has also been described for other proteins containing extensive intrinsically disordered regions [39].

***Interaction with chaperones and transport proteins***. B-domain numerous N-glycosylated residues allow it to bind calnexin, calreticulin, and Golgi receptors, which aids transport and prevents aggregation of intermediate FVIII forms [39].

***Regulation of FVIII interactions in plasma***. Residual B-domain sequences present in circulating inactive FVIII are thought to limit its premature interactions with platelets and inactivating proteases [39]. Makogonenko et al. also reported that the B-domain limits FVIII’s interaction with the heparan sulfate proteoglycans (HSPG) clearance receptor. B-domain deletion exposes the heparin-binding site independently of vWF, thereby promoting FVIII interaction with HSPGs and potentially accelerating its clearance. In contrast, the B domain does not affect FVIII binding to low-density lipoprotein receptor-related protein (LRP) [44]. Conversely, N-linked glycans of the FVIII B domain are thought to be recognized by the carbohydrate-recognition domains of the asialoglycoprotein receptor (ASGPR), potentially contributing to FVIII catabolism in vivo [45].

Although the B-domain is not required for coagulant activity, a subset of B-domain missense variants listed in the CHAMP database is associated with mild HA [46]. Detailed analysis shows that many of these variants are in fact rare polymorphisms that retain 40–150% of normal FVIII activity. Instead, some of these phenotypes arise from co-occurring mutations in conserved domains or through the development of inhibitory anti-FVIII antibodies [37,47,48]. However, several substitutions reduce functional activity below 40%. p.Asn1460Lys and p.Ser1688Leu reduce the specific activity without affecting its expression, whereas the variants p.Glu1057Lys, p.His1066Tyr, and p.Asp845Glu result in a reduced level of functional protein in both the lysate and the conditioned media of Hek293T cells. A further set of substitutions (p.Glu1057Lys, p.His1066Tyr, p.Arg1126Tyr, p.Arg1329His, p.Gln1336Lys, p.Leu1481Pro, p.Ile1698Thr) also impair FVIII thermostability. The authors proposed that R1329H, which lies within the RXXR furin-recognition motif, is pathogenic by disrupting intracellular processing [37], although an earlier study showed that removing this site can instead improve secretion of the single-chain (SC) form [49].

#### 2.2.3. FVIII in the ER and Golgi Apparatus

**Figure 3:**
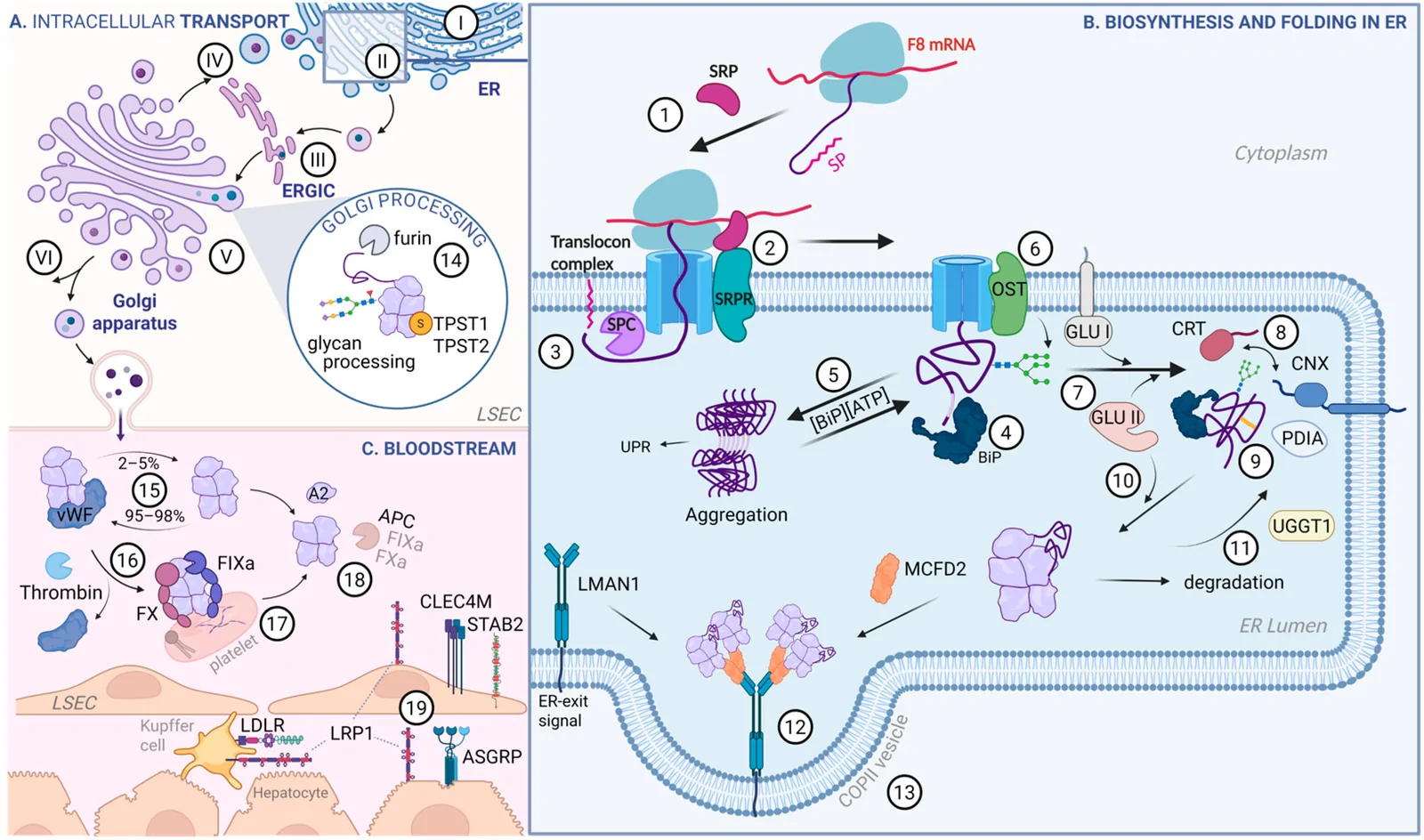
Factor VIII life cycle. (**A**)—Transcription, RNA processing, and nuclear export. (**B**)—FVIII biosynthesis and folding: (1) The SP binds SRP upon emerging from the ribosomal tunnel. (2) The ribosome–nascent-chain–SRP complex binds the ER SRP receptor and is loaded onto the translocon; the SP inserts into the membrane, and FVIII is translocated into the ER lumen. (3) The SP is cleaved by the signal peptidase complex (SPC). (4) BiP retains FVIII in the ER lumen and promotes folding. (5) BiP deficiency causes reversible FVIII aggregation and is associated with the unfolded protein response (UPR). (6) Initial N-glycosylation is mediated by the OST complex. (7) Glucosidases I and II remove the first and second glucose residues, respectively. (8) Calnexin (CNX) and calreticulin (CRT) bind FVIII and mediate its folding cycle. (9) PDI-family proteins and cyclophilin B promote disulfide-bond formation, disulfide rearrangement, and FVIII folding. (10) Glucosidase II removes the final glucose residue, terminating the FVIII-CNX/CRT interaction cycle. (11) UGGT reglucosylates misfolded FVIII glycans, returning FVIII to the CNX/CRT cycle. (12) MCFD2 binds FVIII and, together with LMAN1, forms an ER-export complex. (13) LMAN1 recruits proteins required for COPII-vesicle assembly. III. Properly folded FVIII is transported to the Golgi apparatus through the ER–Golgi intermediate compartment (ERGIC). IV. COPI vesicles mediate retrograde transport of LMAN1–MCFD2 from ERGIC to the ER. V. Golgi processing. (14) B-domain proteolysis, N- and O-glycan maturation, and Tyr sulfation occur in the Golgi apparatus. (**C**)—Circulatory Pathway: VI. Post-Golgi and endosomal–lysosomal trafficking sort FVIII between secretion, degradation, and retrograde-trafficking pathways. (15) In circulation, FVIII is dynamically associated with vWF. (16) Thrombin activates FVIII to heterotrimeric FVIIIa, releasing vWF. (17) FVIIIa and FIXa bind phosphatidylserine and proteins on activated platelets, forming the intrinsic Xase complex. (18) FVIIIa is inactivated by A2-domain dissociation or by APC, FIXa, and FXa. (19) FVIII clearance by receptors on hepatocytes, macrophages, and LSECs, either directly or as a complex with vWF.

***Signal peptide and translocation***. To date, the molecular machinery responsible for the co-translational ER translocation of nascent FVIII has not been defined experimentally. Since FVIII is secreted, translocation is assumed to involve the conserved protein-conducting channel complex Sec61, possibly together with the translocon-associated factors TRAM, TRAP, Sec62/Sec63, and the luminal chaperone BiP (Figure 3). The elements that contribute to FVIII translocation have not been established and remain to be determined [20,50].

FVIII biosynthesis begins with the N-terminal SP (MQIELSTCFFLCLLRFCFS). The hydrophobic core of secretory proteins is expected to be recognized by the signal recognition particle (SRP) as it emerges from the ribosomal exit tunnel. SRP transiently attenuates translation and delivers the ribosome–nascent-chain complex to the SRP receptor at the ER membrane, where it engages the Sec61 translocon, which inserts the hydrophobic segment into the membrane and threads the rest of the peptide through the translocon channel into the ER lumen as it is synthesized. SP cleavage is expected to occur when the Cys–Phe–Ser motif, which contains small uncharged residues at the −3 and −1 positions, reaches the active site of the signal peptidase complex (SPC) located near the luminal exit of the Sec61 translocon. Following cleavage, the mature FVIII polypeptide is released into the ER lumen, where its synthesis, folding, and early post-translational processing continue. The cleaved SP remains membrane-associated and is subsequently degraded [50,51,52].

***Folding and interaction with chaperones in the ER***. Taken together, the evidence points away from cooperative folding of the entire FVIII chain and more towards modular folding followed by docking of the domains. In the N1922S variant, for example, a substitution in the A3 domain disrupts the structure of both A3 and the adjacent C1 domain, while A1, A2, and C2 remain structurally intact [53]. Synthesis of FVIII as a single continuous polypeptide is not essential for the formation of a functional heterodimer: numerous studies have shown that separate delivery of the HC and LC can still yield active FVIII, despite unequal secretion of the two chains [54]. Mah et al. report that co-expressing the two chains within a single cell, with intracellular docking in the ER, is necessary for functional FVIII [55]. However, FVIII activity has also been reconstituted in vitro by reassociation of purified HC and LC after secretion [56]. In addition, the A2 domain, when expressed separately from the remainder of the molecule, can associate with complementary FVIII fragments and restore functional FVIII activity, both upon co-expression with other FVIII domains and following extracellular addition of purified A2 protein [57].

During translocation, A1-A2-B-A3-C1-C2 undergoes a series of post-translational interactions with chaperones, as well as glycosylation and disulfide-bond formation. To stabilize the soluble, aggregation-prone, but capable of correctly folding FVIII, the ER-luminal chaperone BiP (GRP78/HSPA5) binds to hydrophobic regions located predominantly within the A1 domain. BiP assists folding by cycling between high- and low-affinity substrate binding states through cooperative ATP-dependent conformational changes in its ATPase domain, which are further regulated by ER co-chaperones and nucleotide-exchange factors. BiP also retains FVIII at the translocon channel exit within the ER lumen, preventing its return to the cytosol [58,59]. The depletion of BiP leads to FVIII accumulation, impaired disaggregation, and induction of Unfolded Protein Response (UPR) pathways through eIF2α and CHOP [59].

The transmembrane multisubunit oligosaccharyltransferase complex (OST) mediates the initial transfer of Glc_3_Man_9_GlcNAc_2_ from the lipid donor dolichol pyrophosphate-linked donor to asparagine residues within FVIII’s consensus N-X-S/T motifs, in which X is not proline. Glycan transfer occurs as soon as the N-X-S/T site passes through the translocon channel pore, though it can also occur post-translationally at exposed external regions [60].

Glycan attachment is followed by interaction with the luminal glucosidases I and II. Glucosidase I removes the first glucose residue from the α-1,2-linked glucose chain of Glc_3_Man_9_GlcNAc_2_, giving Glc_2_Man_9_GlcNAc_2_, and glucosidase II removes the remaining, yielding the mono-glucosylated glycan, resulting in the monoglucosylated Glc_1_Man_9_GlcNAc_2_. The appearance of this form signals FVIII entry into the calnexin/calreticulin lectin-chaperone cycle, which is critical for correct FVIII folding, prevention of aggregation, and progression through the secretory pathway [59,61]. Calnexin (CNX), a membrane lectin, and calreticulin (CRT), a soluble luminal protein, both recognize the monoglucosylated N-glycan Glc_1_ManₙGlcNAc_2_ on the substrate. The resulting complex anchors FVIII molecules, slowing their trafficking kinetics and allowing them to reach a stable conformation before leaving the ER [61]. Inhibiting the calnexin–calreticulin cycle increases FVIII aggregation without altering its expression level [59]. Calreticulin, however, is also known to bind non-glycosylated hydrophobic peptides, so the mechanism of interaction has not been fully resolved [62].

Efficient protein folding in the ER depends on the cooperative action of chaperones within a single quality-control system [62]. CNX and CRT not only retain FVIII but also recruit further chaperones. FVIII has been shown experimentally to interact with several key components of this network, BiP, CNX, UGGT1, SEL1L, and PDIA6, confirming that it follows the typical chaperone-mediated folding cycle [59]. Calnexin carries a lectin pocket for oligosaccharide binding and a separate domain that interacts with ERp57, which catalyzes disulfide-bond isomerization during protein retention [61,62]. Other isomerases, several PDI-family proteins, cyclophilin B, and ERp29, respectively, contribute to disulfide-bridge formation and rearrangement, carry out peptide-bond isomerization, and provide a general chaperone function [62].

Glucosidase II then removes the last glucose residue from the N-linked glycan, at which point the glycoprotein can no longer bind CNX/CRT, completing the first round of the folding cycle. If the protein has not yet reached its native conformation, it is further modified by the glucosyltransferase UGGT (UDP-glucose glycoprotein glucosyltransferase), which reattaches a single glucose residue to the glycan. UGGT’s TRXL2 and TRXL3 domains are thought to recognize misfolded regions of the polypeptide, targeting reglycosylation to regions where hydrophobic patches remain exposed [62].

Recent studies point to a role for CALR distinct from that of CNX in interacting with various substrates and signaling pathways both inside and outside the ER lumen. CALR has not been implicated in proteasomal degradation but does interact directly with GABARAP, which colocalizes with FVIII and with the autophagosomal protein VAMP8 in the cell. It remains to be established what role CNX/CALR plays in maintaining cellular homeostasis and autophagy via ER-phagy receptors such as FAM134B and GABARAP [61].

***Post-folding stage and export from the ER***. Following N-glycan trimming by glucosidases I and II in the ER, correctly folded FVIII enters the conventional secretory transport pathway to the Golgi apparatus (GA) via the ER–Golgi intermediate compartment (ERGIC) [63]. FVIII, however, has no membrane domain or ER-exit signal of its own, and so cannot be efficiently incorporated into vesicles independently. Efficient export instead requires the LMAN1–MCFD2 cargo-receptor complex [64], which concentrates FVIII within forming COPII vesicles [65,66].

LMAN1 (ERGIC-53) is a type 1 transmembrane protein of the lectin family that, besides its transmembrane domain, carries a large luminal head that binds glycans. This head region is positioned at a considerable distance from the membrane and is connected to it by a long, flexible stalk. A recently resolved cryo-EM structure of the LMAN1–MCFD2 complex showed that LMAN1 is in fact a tetramer, not a hexamer as previously thought, and that it binds MCFD2 with 1:1 stoichiometry, one MCFD2 molecule per protomer [64,67,68,69]. The tetramer itself is held together by interchain disulfide bonds so that oligomerization persists both before and after FVIII binds. Each LMAN1 subunit contains a carbohydrate-recognition domain (CRD), two α-helices (S-H1 and S-H2), and connecting loops that give it the flexibility to accommodate ligands of different positions and lengths [67]. Beyond its transport role, LMAN1 can also transiently bind correctly folded glycoproteins within the ER lumen, helping glucosidase II remove the terminal glucose. This may allow certain proteins to exit the ER quality-control cycle slightly early, before glucose trimming is fully complete [70].

MCFD2 is a soluble, monomeric luminal protein of around 16 kDa, carrying two Ca^2+^-binding EF-hand domains that mediate its interaction with both LMAN1 and FVIII [71]. Recent data show that MCFD2 also binds substantial amounts of Zn^2+^, which may interfere with the LMAN1-binding site, which is masked by the N-terminal helix of Zn^2+^-bound MCFD2, apparently giving it a regulatory role in modulating substrate binding and release [67].

The LMAN1–MCFD2 interaction within the membrane complex is mediated by N-terminal elements of LMAN1 and the two Ca^2+^-binding EF-hand domains of MCFD2 [70]. Beyond its adaptor role in FVIII binding, this interaction is also needed to return MCFD2 to the ER as the complex cycles between the ER and ERGIC [66]. LMAN1 mutations in its carbohydrate-binding regions disrupt its interaction with FVIII without affecting MCFD2 binding [69], and FVIII binding to LMAN1 on its own is not enough to retain FVIII stably on the membrane [64]. MCFD2 is therefore likely the main FVIII-interacting partner for retention of LMAN1, with LMAN1 acting as a carrier for MCFD2 [64,72]. MCFD2 that can no longer interact with LMAN1 can still bind FVIII, showing that its proposed Ca^2+^-induced folding is not required for this function [72], though in this case FVIII is no longer secreted efficiently [64].

In the context of FVIII engineering, this raises the question of whether the heavily glycosylated regions of the B-domain are actually necessary for efficient secretion. LMAN1 possesses structurally and functionally characterized mannose-binding sites, and experiments in which FVIII glycosylation was inhibited showed that the loss of N-glycans impaired its binding to LMAN1 [70]. It later emerged, however, that this interaction is not strictly required for FVIII secretion, as overexpressing MCFD2 is sufficient to restore FV and FVIII secretion in LMAN1-deficient cells [64]. Conversely, other experiments have shown that exogenously expressed LMAN1 mutants defective in mannose binding can still rescue most of the FVIII secretion defect in LMAN1-knockout cells [64]. Earlier confocal microscopy studies had already noted that removing the B-domain does not alter FVIII’s transport route out of the ER [63]. However, the transport mechanisms of other glycoproteins that share the same transport machinery may differ from those of FVIII. LMAN1’s interaction with α1-antitrypsin (AAT) and cathepsin Z, for instance, does not require MCFD2 [64], and inhibiting glycosylation reduces secretion of these proteins far more markedly [73], supporting the diversity of cargo-recognition mechanisms even within a single receptor complex. LMAN1 may therefore interact with FVIII through a lectin-independent, direct protein–protein interaction, analogous to its interactions with transmembrane clients [67].

One study reports that MCFD2 ligands share a common motif, SDLL(-/M)LL(K/R)QS, which mediates specific binding to the cargo receptor complex [74]. In FVIII, the corresponding sequence is SDLLMLLRQS, located at residues 807–816 of the B domain. A subsequent study, however, found that deletion of this sequence or alanine substitution of its residues did not reduce FVIII activity or the amount of FVIII secreted by HEK293T, HepG2, or HCT116 cells [64]. Rational engineering of FVIII will therefore need further structural studies of the transmembrane complex-ligand interaction to establish the true binding sites.

***Current understanding of the mechanism of FVIII ER-Golgi transport***. MCFD2 functions as a module for FVIII capture and for maintaining its stable association with LMAN1. It binds FVIII in the ER lumen, while the interaction with LMAN1 is further strengthened by additional recognition of glycan structures, thereby forming a stable ternary complex that supports ER exit [64,70]. In turn, LMAN1 anchors and concentrates FVIII at the luminal face of the ER membrane and contains a canonical ER-exit signal (diphenylalanine motif) in the cytosolic C-terminal tail of its transmembrane domain [65]. In the standard export mechanism, the LMAN1 signal sequence recruits the SEC23/24 heterodimer to the membrane surface, which in turn recruits the SEC13/31 heterotetramer. As the SEC13/SEC31 heterotetramers bind successively to SAR1-GTP–SEC23/SEC24 complexes on the membrane of the transitional ER at ER exit sites (ERES), they promote the assembly of the COPII coat into a lattice, producing a COPII vesicle (50–200 nm) [65,70]. FVIII colocalizes with COPI markers [61,63]; however, there is no direct evidence that it is actually packaged into COPI vesicles and returned to the ER. Notably, its colocalization with COPI is more pronounced than with COPII.

The classical view holds that LMAN1 releases its substrate in a calcium-dependent manner, since LMAN1 binds high-mannose glycans in a Ca^2+^-dependent way. This model fits a direct FVIII–LMAN1 interaction via mannose residues and agrees with the substantially lower Ca^2+^ concentrations found in the ERGIC and Golgi compared with the ER; complex stability, meanwhile, has been shown not to depend on pH across the physiological range found in the ER, ERGIC, and Golgi [70]. LMAN1 and MCFD2 are likewise retained in a Ca^2+^-dependent manner, but their complex is more stable and persists even at reduced Ca^2+^ concentrations [70,71], so while a drop in ERGIC luminal Ca^2+^ may disrupt FVIII binding, the LMAN1–MCFD2 receptor complex itself does not dissociate over several cycles between the ER and ERGIC/cis-Golgi. Repeated interaction with the cargo receptor and retrograde traffic of the released FVIII may instead be prevented by a change in glycan profile, since Golgi mannosidases can rapidly cleave the α-1,2-linked mannose groups on cargo proteins [70]. For FVIII, however, whose glycan-mediated binding is of secondary importance, it is more likely that once FVIII is released, the binding site is blocked by the N-terminal helix of MCFD2 in response to Zn^2+^ binding, consistent with the higher Zn^2+^ concentrations found in the Golgi relative to the ER and other organelles [67].

Dysfunction of the LMAN1–MCFD2 complex causes combined FVIII/FV deficiency, with activity retained at 5–30% [39,64], underlining the importance of these receptors without making the transport mechanism strictly indispensable. Evidence continues to accumulate for alternative FVIII secretion pathways: across various cell lines, LMAN1 deficiency reduces FVIII secretion to varying degrees [64], and in mice, double knockout of LMAN1 and MCFD2 produced higher FVIII levels than LMAN1 deficiency alone [75]. An excess of the MCFD2–FVIII complex may be enough on its own to exit the ER via bulk flow [64]; other tissue-specific luminal lectins have also been described, VIP36 (LMAN2), VIPL (LMAN2L), and ERGL (LMAN1L), which may serve auxiliary or compensatory roles alongside LMAN1 [67].

FVIII’s colocalization with Rab8, VAMP8, and Rab7 points to the involvement of post-Golgi transport and the endosomal–lysosomal compartments, with the protein actively sorted between secretion, degradation, and retrograde traffic. Under stress, FVIII may be redirected towards degradation or recycling via lysosomal and autophagosomal mechanisms [61]. Overexpressing LMAN1 and MCFD2 together does not further increase FVIII secretion, so the amount of LMAN1–MCFD2 receptor complex is not what limits the rate of FVIII transport through the ER–Golgi [64]. Correctly folded and processed FVIII moves rapidly through the secretory pathway, whereas misfolded FVIII fails to be secreted even after 10 h [63]. It remains unclear what proportion of the protein reaching the trans-Golgi is actually secreted, whether this secretion is constitutive, or whether FVIII is held within the cell until a release signal arrives.

***Glycosylation***. The most common N-glycans on FVIII are of the complex type, although high-mannose and hybrid forms also occur, with the glycosylation profile dominated by biantennary N-glycans and tri- and tetra-antennary types occurring less often [76]. All N-glycans share the common core structure GlcNAc_2_Man_3_, onto which mannose, galactose, N-acetylglucosamine, N-acetylgalactosamine, sialic acid, and fucose are added in linear and branched arrangements [77]. The complex-type-*N*-glycans of plasma FVIII are mainly characterized by core fucosylation and extensive antenna coverage with either sialic acids (66%) or ABO(H) blood-group antigens (10%).

Each glycan undergoes specific, stepwise maturation as FVIII moves along the secretory pathway, driven by numerous ER and Golgi glucosidases and glycosyltransferases [76].

FVIII carries 25 potential N-glycosylation sites: 2 in the A1 domain, 1 in A2, 19 in the B-domain, 1 in a3, 1 in A3, and 1 in C1 [39,76]. Not all sites are occupied, however, since folding during synthesis in the ER lumen makes some of them inaccessible to the oligosaccharide precursor: as a result, the sites in A2 and a3, along with 2–3 sites in the B-domain, remain non-glycosylated in native full-length FVIII. This also explains the observed heterogeneity of glycoforms: because the A2 and C1 sites are less exposed, their modifications are restricted to hybrid and short, non-fucosylated complex-type N-glycans [76,77].

FVIII O-glycosylation, formed via a bond between O-GalNAc and serine or threonine, has attracted less study. One O-glycosylation site has been found in the A2 domain, along with 8 peptide fragments within the B-domain whose O-glycan positions remain undetermined. Most O-glycoforms are Core 2 structures, with the core trisaccharide GlcNAc-β1,6-(Gal-β1,3-)GalNAc; a Core 1 variant is also common, the tetrasaccharide disialyl-T antigen, with the fucosylation and sulfation modifications characteristic of plasma FVIII [40].

Together, glycosylation shapes FVIII’s folding, transport, and half-life. The glycosylation profile is tissue- and organ-specific, and disrupting it can lead to protein degradation rather than secretion [39,77]. Interpreting the functional role of individual glycans is complicated, however, by how closely their influence is tied to that of the FVIII B-domain’s a.a. sequence, which carries the great majority of the protein’s N- and O-glycosylation sites [42].

***Trans-Golgi FVIII Maturation.*** Tyrosine sulfation is a late modification of secreted proteins in the trans-Golgi. It is catalyzed by the resident Golgi enzymes TPST1 and TPST2, which transfer sulfate from 3′-phosphoadenosine-5′-phosphosulfate (PAPS) to the phenolic hydroxyl group of Tyr. The modification is determined by a locally acidic, Asp/Glu-rich environment, Tyr accessibility, and the residence time of the protein in the late Golgi [78]. In FVIII, Tyr346, Tyr718, Tyr719, Tyr723, Tyr1664, and Tyr1680 are sulfated. Sulfation of Tyr346 and Tyr1664 promotes efficient thrombin-mediated activation and cleavage of FVIII. The Tyr718/Tyr719/Tyr723 cluster supports intrinsic tenase-complex assembly and enhances intrinsic Xase activity, whereas sulfated Tyr1680 is particularly important for high-affinity binding to vWF [79]. Inhibition of cellular sulfation did not markedly suppress FVIII synthesis or secretion but reduced its procoagulant activity by approximately 5-fold. Thus, cells can secrete FVIII with incomplete sulfation occupancy; however, this product interacts less efficiently with vWF, is activated less efficiently by thrombin, and has reduced functional activity [80].

Furin preferentially cleaves luminal secretory cargoes at multibasic motifs, most commonly R-X-(K/R)-R, under the mildly acidic, Ca2+-containing conditions of the late Golgi/trans-Golgi network [81]. Furin cleaves the B-domain/a3 junction, principally at Arg1648, thereby separating FVIII into a heterogeneous heavy chain and the a3-A3-C1-C2 LC. The chains remain non-covalently associated, partly through divalent-metal-ion-dependent interactions, and FVIII is secreted predominantly as a heterodimer [82]. Furin may not be the only proprotein convertase able to process this region of FVIII. In recombinant-expression studies, PCSK7 and PCSK6/PC5/6 also showed activity toward the Arg1648 site. PCSK6 activity was observed particularly in an overexpression context. Therefore, furin processing is best interpreted as processing by a furin-family convertase network whose relative contribution depends on cell type, expression level, secretory-pathway localization, and FVIII construct context [83].

### 2.3. FVIII Structure in the Bloodstream and Inactivation

#### 2.3.1. vWF Partnership from Trafficking to Clearance

Von Willebrand factor is a large, multidomain glycoprotein of ~300 kDa. Once secreted into plasma, it exists as multimeric complexes of dozens of subunits and can circulate freely or remain anchored to cell membranes. vWF is the plasma partner of non-activated FVIII, with which it forms a stable circulating complex [84,85]. This binding shields FVIII from premature interaction with phospholipid membranes, from inactivating proteases acting on its light chain, and from clearance proteins, making vWF a key stabilizer of FVIII in the bloodstream [86,87]. vWF is also thought to act as a spatiotemporal regulator of activity, concentrating FVIII at sites of injury. The relationship runs both ways: FVIII in turn modulates ADAMTS13-mediated proteolysis of vWF, forming a negative feedback loop so that as FVIII levels rise, cleavage of vWF multimers increases, helping maintain hemostatic balance [88]. The FVIII–vWF interaction is marked by pico- to nanomolar affinity and strong evolutionary conservation, particularly in the directly contacting D′D3 domain of vWF and C1 domain of FVIII, pointing to the evolutionary importance of this complex and strong negative selection against mutations that would disrupt it [89].

Exactly how and where FVIII and vWF physically associate in vivo remains unclear. Vascular endothelial cells are the main source of circulating vWF and store it in specialized organelles called Weibel–Palade bodies (WPBs). Megakaryocytes also synthesize vWF, which is stored in platelet α-granules. LSECs may produce vWF, leaving open the possibility of intracellular association with FVIII, but exhibit low vWF expression [90]. Natively expressed FVIII in endothelial GMVEC and HUVEC cells colocalizes with vWF in WPBs and is released, along with their contents, in a regulated fashion that coats membrane-anchored ultra-large VWF strings (ULVWF) [91]. The intracellular FVIII–VWF binding sites, which govern their joint transport, likely differ from what is needed to assemble the complex in circulation: variants of VWF type 2N, whose affinity for FVIII in circulation is substantially reduced, still direct FVIII to WPBs intracellularly [92]. It also remains unknown whether FVIII bound to VWF strings keeps its association with smaller vWF cleavage products after ADAMTS acts on them, dissociates instead, or stays complexed with ULVWF right up to the point of activation [91].

Storage of FVIII within vWF is secondary to vWF’s own storage, and the association is not required for the two proteins to traffic together or for vWF’s regulated storage [93]. When FVIII is transfected into non-endothelial cells (HT-1080, HepG2), it stays in the cytoplasm and does not form WPB-like structures [94], though this appears to be cell-type dependent, since expressing it in a vWF knockout megakaryocyte model led to FVIII being stored in α-granules instead [86,93].

In normal plasma, 95–97% of FVIII circulates within the vWF complex (Kd = 0.2–1.5 nM), which provides the main protection against clearance by masking the recognition sites that clearance proteins would otherwise bind [84,85]. The remaining 3–5% circulates as free FVIII, with a half-life of ~2 h; vWF itself circulates for ~20 h, and the close relationship between FVIII and vWF levels suggests the remaining FVIII is cleared as part of the vWF complex [86]. Even though most FVIII sits within the FVIII–vWF fraction, the system remains in dynamic, reversible equilibrium with free FVIII [95]. Accounting for both pathways, FVIII’s overall half-life is therefore around 12 h [96,97], and Swystun et al. calculate that this implies roughly 24% of FVIII is cleared from the bloodstream in its free form even before activation [98].

Although each vWF monomer is capable of binding FVIII, the FVIII–vWF complex in the bloodstream maintains a stable stoichiometry of around 1:50 [86]. The interaction is held together mainly by electrostatic forces, and the balance of a high association rate with relatively fast dissociation protects FVIII from degradation while still allowing its rapid release on thrombin activation [85]. The FVIII–vWF interaction site lies entirely within the FVIII light chain, with the A3/C1 domain surface and the a3 linker playing the key role, binding the D′D3 domain of vWF [86]. Residue Y1680 of the FVIII a3 peptide interacts with R816 of vWF, while nearby acidic residues (D1676, D1678, and D1681) form salt bridges with R820, R826, and R768 [35,87]. The D3 domain of vWF wraps around the base of both C domains, making intermolecular contacts through hydrophobic loops that also interact with membrane surfaces. Binding triggers large conformational changes in vWF, while FVIII’s own structure remains almost unchanged [35].

Fuller et al. propose a two-step binding model: high-affinity attachment of vWF’s D′-TIL′ to A3/a3/C1, followed by an ~85° rotation of vWF’s D3 domain that blocks the C1/C2 membrane-binding regions [87]. Removing the N-terminal acidic region of the FVIII light chain during activation cuts FVIIIa’s affinity for vWF roughly 1400-fold, releasing FVIIIa from the complex [86]; the a3 peptide may still be held by D′D3, but the remaining FVIII–vWF interactions outside FVIII-a3 are not strong enough to offset the entropic cost of VWF-D′D3’s restricted mobility, leading it to dissociate from FVIII [87].

#### 2.3.2. Thrombin Activation

Thrombin activates FVIII by cleaving three arginine residues, Arg372 and Arg740 in the heavy chain and Arg1689 in the light chain, releasing acidic peptides in the process [35,99]. The FVIII heterodimer (in complex with vWF) first engages thrombin through rapid electrostatic contacts between thrombin’s anion-binding exosites and FVIII’s acidic surroundings, which then position the arginine residues at the catalytic site [100].

Proteolysis proceeds at different rates at the three sites. Besides competing for thrombin’s active site, the kinetics are shaped by the a.a. composition flanking each cleavage site, reflecting how strongly hydrogen bonds and nonpolar contacts stabilize the flexible FVIII loops there. This produces the overall cleavage order Arg740 > Arg1689 > Arg372. Proteolysis at Arg372 can be sped up 10-fold, and at Arg740/Arg1689 3-fold, by optimizing the flanking residues, though tampering with these regions risks disrupting FVIII function [101]. Each site plays a distinct role: cleaving Arg1689 releases FVIII from vWF, Arg740 has only minor functional significance, and the rate-limiting Arg372 exposes the FIXa-binding site in the A2 domain (Tyr555–Asp569) according to MD simulations, triggering cofactor activity [102,103]. Proteolytic activation also increases A2 domain mobility, and its eventual dissociation limits how long FVIIIa’s coagulant activity lasts [104].

An alternative activation pathway also exists, involving FXa within the TF–FVIIa–FXa complex, where FVIII is cleaved at the same three sites independently of thrombin [105].

#### 2.3.3. Membrane Binding

Upon vascular injury, platelets become activated and initiate primary hemostasis by acquiring a proaggregatory phenotype. This process triggers the formation of a contracting platelet plug that serves as the first physical barrier to bleeding. Concurrently, a subset of platelets transitions from a proaggregatory to a procoagulant phenotype, characterized by increased exposure of phosphatidylserine (PS) on the outer membrane leaflet [106].

The current model of FVIII binding to the surface of activated platelets is based on its interaction with anionic phospholipids, primarily PS. This binding occurs with high affinity (Kd 1–5 nM) and is mediated by the C1 (residues 2021–2170) and C2 (residues 2171–2332) domains, which adopt β-sandwich structures with protruding β-hairpin loops [107]. Following activation of FVIII to FVIIIa, its affinity for PS-containing membranes increases further, with the dissociation constant decreasing from approximately 5.0 ± 0.6 nM to 0.65 ± 0.19 nM, primarily due to a reduced dissociation rate of the complex relative to inactive FVIII [108].

The high local concentration of membrane-bound FVIIIa enables efficient assembly of the FVIIIa–FIXa tenase complex, while the presence of PS increases the catalytic efficiency of this complex by approximately 1500-fold [109].

However, recent findings indicate that this model does not fully explain FVIII binding to the platelet surface under physiological conditions. Thrombin-activated platelets expose up to 4% PS in the outer membrane leaflet, whereas the number of FVIII-binding sites substantially exceeds this capacity. Therefore, FVIII binding cannot be explained solely by its interaction with phosphatidylserine. In experiments with lactadherin, a competitive PS-binding protein, less than 10% of platelet-bound FVIII was displaced. Moreover, an FVIII variant with impaired PS-dependent binding retained the ability to interact with activated platelets [110].

These observations led to the hypothesis that surface fibrin (SF), associated with αIIbβ3 integrin, represents the principal physiological receptor for FVIII. FVIII was shown to bind SF with an affinity comparable to that observed for platelet binding, while the C2-domain-directed antibodies ESH8 and ESH4 efficiently inhibited FVIII binding to SF and suppressed more than 90% of platelet-dependent FVIII activity, whereas activity in model PS-containing vesicles was reduced by only approximately 35% [106,109,110]. These results suggest that, under physiological conditions, FVIII recruitment to the surface of activated platelets is determined not only by interactions with PS but also by fibrin-derived protein-binding sites, with both mechanisms likely contributing to the efficient localization and function of the intrinsic tenase complex at the site of clot formation.

#### 2.3.4. Xase Complex

The intrinsic tenase (Xase) complex is formed following thrombin-mediated activation when FVIIIa binds to the surface of activated platelets. The complex assembles on an anionic phospholipid membrane and comprises the protease FIXa, the cofactor FVIIIa, and the substrate FX [111]. Assembly of the complex begins with the binding of FVIIIa and FIXa to anionic phospholipid surfaces of platelets. Although FIXa possesses intrinsic, FVIII-independent membrane affinity through its Gla domain, FVIII binds phospholipids with several-fold higher affinity (Kd 0.2–2 nM to Kd 10 nM). Thus, FVIIIa serves as a high-affinity membrane anchor for FIXa. In addition, phospholipids further increase the affinity of FVIIIa and FIXa for each other by approximately one order of magnitude [112]. The FVIIIa–FIXa interface extends along FIXa and involves the A2-a2 and A3-C1-C2 domains of FVIIIa. Structurally, the catalytic domain of FIXa interacts with the A2 domain of FVIIIa, whose acidic C-terminal tail anchors FVIIIa to FIXa through electrostatic interactions with FIXa exosite II, formed by three helices of the FIXa catalytic domain that constitute part of the principal interface; meanwhile, the light chain of FIXa wraps around the A3-C1 domains of FVIIIa to optimize binding to the lipid membrane [35,113,114]. Free FVIIIa, in the absence of FIXa, exhibits an approximately 20° bend at the junction between the A and C domains; this conformation is lost upon FIXa binding and appears to be incompatible with assembly of the active tenase complex [35].

FVIIIa binds intact zymogen FIX with more than 10-fold higher affinity than activated FIXa, allowing FIX to competitively displace FIXa and form an inactive “pro-Xase” complex. Given the high plasma concentration of the zymogen (approximately 90 nM versus approximately 0.7 nM FVIII), this interaction may buffer coagulation until a sufficiently strong local signal enables complete FIX activation [115,116].

Binding of FX to the FIXa–FVIIIa enzyme–cofactor complex is a multistep process that reduces the Km for FX activation from 1700 nM in free solution to 28 nM on the membrane in the presence of the cofactor; thus, complex formation with FVIIIa increases the affinity of FIXa for its substrate [117]. The excess of FX in vivo suggests that intrinsic Xase complexes are saturated with substrate under physiological conditions [113].

Thus, FVIIIa functions as a crucial cofactor, increasing the catalytic activity of FIXa by 10^4^–10^6^-fold when assembled into a membrane-bound complex relative to FIXa in free solution [112,113,117]. The increase in activity is explained by exposure of the FIXa active site through a conformational change in FIXa’s autoinhibitory 99-loop, adjacent to the substrate-binding pocket, following binding to FVIIIa [35].

#### 2.3.5. Inactivation

Inactivation of the Xase complex is governed by two main processes: dissociation of the A2 subunit and proteolytic cleavage by activated protein C (APC) [35]. In the isolated state, without binding partners, purified FVIIIa is inactivated within approximately 6 min because of spontaneous dissociation of the A2 subunit from the A1/A3-C1-C2 heterotrimer (Kd ~500 μM) [118]. When FVIIIa is incorporated into a complex with FIXa, its structure is stabilized, and A2-subunit dissociation is slowed by up to 10-fold compared with free FVIIIa. In vitro, APC-mediated inactivating proteolysis occurs over approximately 1 h and therefore makes a secondary contribution. APC selectively inactivates FVa and FVIIIa by cleavage at two sites: Arg562, which results in release of the A2 domain, and Arg336, located immediately upstream of the C-terminal acidic region of the A1 subunit. These cleavages disrupt FVIIIa-dependent modulation of the FIXa active site. FIXa binding protects FVIIIa from cleavage at Arg562 [113,119]. It is also known that cleavage at Arg336 occurs approximately six-fold faster than cleavage at Arg562. Cleavage at Arg336 does not completely inactivate FVIIIa; however, the presence of activated cofactors can modulate the rate of APC-mediated cleavage [119].

In addition to APC, FIXa itself and the reaction product FXa can also cleave FVIIIa at Arg336 in the A1 subunit. FXa additionally cleaves Lys36 in A1. This cleavage appears to alter the conformation of the A1 subunit and substantially reduce its affinity for the A2 subunit, although the precise molecular mechanism remains less well characterized [35].

#### 2.3.6. Clearance

FVIII is cleared from the bloodstream by receptors on hepatocytes, macrophages, and endothelial cells. As noted above, clearance proceeds either through direct, rapid interaction with FVIII itself or, more slowly, through capture of the FVIII–vWF complex, and receptors with non-specific binding can engage either partner. Recognition may rest on protein–protein interactions specific to key residues, mainly in the FVIII light chain, or on recognition of surface carbohydrates, mannose residues, or modified sialylated and asialylated N- and O-linked glycans [120].

The key FVIII clearance receptor is LRP1 (Kd ~20 nM), which recognizes clusters of lysine residues in the light chain’s A3, C1, and C2 domains, as well as in the A2 domain. The light-chain sites may be accessible before FVIII binds vWF or after it dissociates, while the A2 site is exposed only after activation, together ensuring selective clearance of all forms of FVIII from circulation [86]. FVIII binding to LRP1 follows the classical model for this receptor: high affinity arises from avidity, as spatially separated lysines engage several CR modules within LRP1’s cluster IV, forming a stable, bivalent complex [121]. Removing the lysines that interact specifically with cluster IV could, in principle, extend FVIII’s half-life, but the variability of LRP1 contacts sustains high affinity through compensation, which may make it difficult to disrupt this interaction by mutating individual lysines alone [122]. The lower-affinity receptors LDLR (Kd ~30–60 nM) and HSPG concentrate FVIII at the membrane and, by binding positively charged residues much as LRP1 does, help capture it even at low plasma levels (≤1 nM). SCARA5 and SCARA1, by contrast, bind protein determinants on vWF with high affinity but do not interact with FVIII at all [120].

Hepatic and endothelial receptors that recognize carbohydrate structures, ASGPR, SIGLEC5, CLEC4M, MGL, and STAB2, bind different FVIII and vWF glycans: ASGPR recognizes desialylated N-glycans (Kd ~2 nM); SIGLEC5 recognizes sialic acids, though within the FVIII/vWF complex it recognizes only vWF; CLEC4M binds mannose-containing N-glycans; MGL binds terminal GalNAc/Gal motifs on B-domain O-glycans; and STAB2 binds glycosaminoglycans [95,120]. Together, these interactions help control the fate of glycosylated FVIII and vWF, including removal of abnormally modified molecules [98]. Despite this diversity of glycan-dependent mechanisms, glycosylation’s overall contribution to FVIII clearance appears limited: FVIII-BDD has the same half-life as full-length FVIII [45,120].

## 3. Synthetic FVIII Design Approaches

### 3.1. A Historical Overview of Shortened FVIII Variants

Treating HA with plasma-purified FVIII from healthy donors carries a risk of transmitting blood-borne infections, creating a clear need for a safer therapeutic option. Advances in understanding FVIII structure, together with cloning of the full-length approximately 7 kb FVIII cDNA in 1984, enabled the development of recombinant FVIII products. The first full-length products to reach the market were Recombinate^®^ (Genetics Institute/Baxter; approved in 1992) and Kogenate^®^ (Bayer; approved in early 1993) [123]. FVIII yields, however, were up to 10,000-fold lower than for proteins of comparable size. This intrinsic manufacturing inefficiency contributed to the high cost of FVIII products and constrained their availability for prophylactic use [124]. To combat low yields, a necessary and functionally justified step was deletion of the FVIII B domain, which increased recombinant FVIII expression in mammalian cell cultures by approximately two orders of magnitude [124]. B-domain’s natural tendency to be released in plasma, its low homology (more than 200 deletions found relative to the porcine orthologue [43]), and its many glycosylation sites, which could potentially prolong intracellular retention [125], together provided the rationale for removing the B-domain [126,127]. The functional equivalence of B-domain-deleted (FVIII-BDD) variants to full-length FVIII, reduced product heterogeneity, and an experimentally confirmed increase in yield drove the widespread adoption of FVIII-BDD variants in manufacturing.

A guiding principle in developing the first BDD variants was to preserve the processing sites critical for FVIII’s native maturation. The earliest experiments already showed that truncating the B-domain substantially raised *F8* mRNA levels (up to 7-fold) without reducing translation efficiency [42,43,126]. Early variants included one with residues 1001–1591 deleted, which retained FVIII’s coagulant activity [43]; the variant VIIIdes-797–1652, which shrank the B-domain from 909 to 142 a.a. while preserving activity, normal thrombin sensitivity, and vWF binding [126]; deletion Δ771–1666 (FVIII delta II), which gave a fivefold rise in expression over full-length FVIII, was secreted predominantly as an SC owing to the absent furin cleavage site and retained normal thrombin activation [128]; and complete removal of the B-domain in the variant des-(741–1668), which showed that, even without several of the classical B-domain processing points, the molecule could still be efficiently cleaved by thrombin and factor Xa, bind vWF, and perform comparably to natural dimeric FVIII in vivo [129].

A pivotal step towards FVIII-BDD was described by Lind et al. [130]. In their work, they tested three approaches in search of a natively processed variant; that is, the variant that retains only the N-terminal part of the B-domain, combines N- and C-terminal regions, or replaces the B-domain entirely with a short insert of one to four arginines. Two variants met the requirements: rVIII-SQ (referred to hereafter as **FVIII-SQ**) and rVIII R5, both carrying the furin recognition motif (Arg at positions −1 and −4 from Glu1649). FVIII-SQ, with its relatively compact 4371 bp cDNA, became the therapeutic molecule of choice for most HA replacement and gene therapies (owing largely to the AAV vector’s limited packaging capacity) and the basis for most subsequent variants with improved properties [130,131,132,133,134,135,136,137]. Clinical data confirmed that Refacto (FVIII-SQ) was safe and carried no increased risk of nAbs formation [132]. Later products developed to the same principle, FVIII-N8 (Novo Nordisk, retaining 10 N- and 11 C-terminal B-domain residues) and h-cl rhFVIII (Octapharma, with a 16-a.a. linker), became the basis for NovoEight, N8-GP, and Nuwiq [138,139].

### 3.2. Optimizing Linker Composition

#### 3.2.1. Elimination of the Furin Processing Site

**Figure 4:**
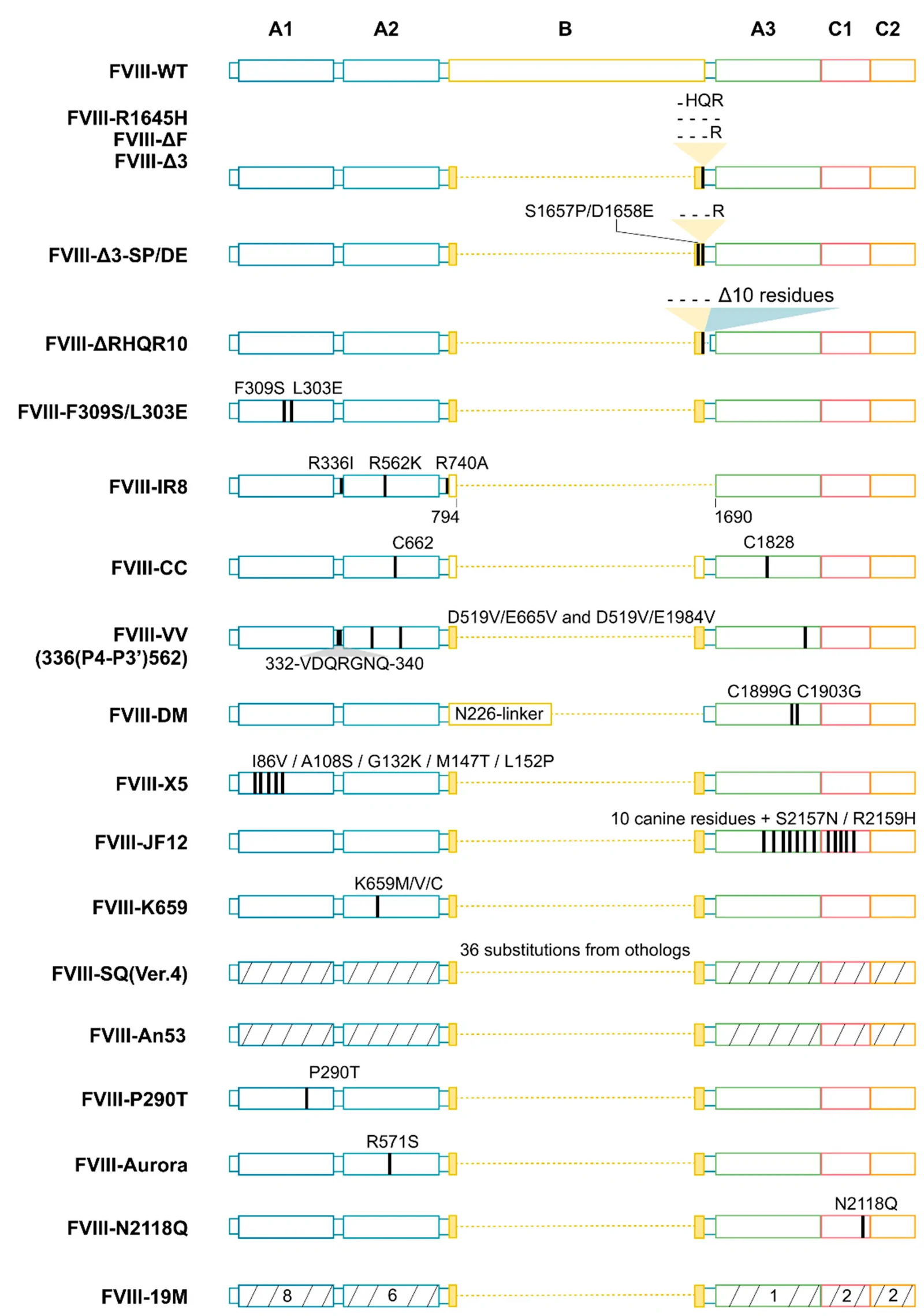
Engineered FVIII variants. FVIII-WT, FVIII-R1645H [49], FVIII-ΔF [141], FVIII-Δ3 [142], FVIII-Δ3-SP/DE [143], FVIII-ΔRHQR10 [144], FVIII-F309S/L303E [145], FVIII-IR8 [133], FVIII-CC [146], FVIII-VV (336(P4-P3’)562 [147], FVIII-DM [148], FVIII-X5 [137], FVIII-JF12 [136], FVIII-K659 [135], FVIII-SQ (Ver.4) [149], FVIII-An53 [150], FVIII-P290T [151], FVIII-Aurora [152], FVIII-N2118Q [153], FVIII-19M [134]. Hatched regions indicate the presence of mutations within the corresponding domains. Numbers inside the domains indicate the number of mutations in each domain. An underscore (_) denotes a single a.a. deletion of the furin-recognition site, RHQR. Δ10 indicates deletion of 10 residues immediately downstream of the furin-recognition site. A yellow-filled rectangle denotes the SQ linker. An unfilled rectangle or a rectangle shown in a different color indicates a linker distinct from the SQ linker.

Comparison of human FVIII with its canine ortholog (cFVIII), which is secreted predominantly as a SC molecule, identified an R1645H substitution in the furin-recognition motif (HHQR). This substitution accounts for the inefficient intracellular cleavage of cFVIII. Introduction of the corresponding substitution into human FVIII (**hFVIII-R1645H**) increased protein secretion (Figure 4) [140]. Later studies confirmed that intracellular furin cleavage generally impairs secretion of FVIII-BDD, prompting a search for optimized variants that bypass this processing step [141]. Removing the RHQR site entirely (**FVIII-ΔF**) raised coagulant activity and increased circulating FVIII levels in vivo after AAV delivery in mouse and dog models, confirming the promise of such constructs for HA therapy [141]. Deletion of two to four a.a. within the RHQR furin-recognition site increased the proportion of SC FVIII from 23% to 48–57%, still allowed thrombin-activating cleavage, and increased secretion of functionally active protein by 1.5- to 3-fold both in vitro and in vivo after AAV delivery, without altering its half-life. The most effective variant was **hFVIII-Δ3**, in which the three residues RHQ (Δ1645–1647) were deleted from the furin-cleavage site [49,142].

However, even after complete removal of the furin-recognition site, a two-chain protein was still generated through an additional intracellular cleavage site downstream of Ser1657 within the acidic a3 linker region (the protease responsible for this cleavage has not been identified) [142]. Further optimization introduced the substitutions S1657P/D1658E, which are conserved in the corresponding linker regions of canine cFVIII (1649-PE-1650) and porcine FVIII (1439-PE-1440) [143]. The final variant, **hFVIII-Δ3-SP/DE**, combining mutations at both sites, was secreted as SC 99% of the time in vitro (compared with 88% for cFVIII) and 82% in vivo, at mRNA levels comparable to hFVIII-SQ. In the HA/CD4KO mouse model, AAV-mediated delivery of hFVIII-Δ3-SP/DE gave a 2-fold rise in protein level (68.5 ng/mL at 4 × 10^12^ vg/kg) and a 5-fold rise (247 ng/mL at 1.2 × 10^13^ vg/kg) by week 8 compared with hFVIII-SQ, consistent with the in vitro findings. In contrast, modification of the first cleavage site alone did not enhance secretion. Codon-optimizing the hFVIII-Δ3-SP/DE sequence produced a 20-fold rise in plasma FVIII:Ag compared with hFVIII-BDD at a dose from 2 × 10^12^ to 4 × 10^12^ vg/kg by week 4. Importantly, the minimal modifications incorporated into the hFVIII variants did not increase immunogenicity relative to wild-type FVIII-BDD during 22 weeks after injection, unlike canine FVIII-BDD [143].

A different research group conducted a study that evaluated 14 deletion variants targeting the RHQR furin-recognition site and its flanking a.a. residues. This construct, in which 10 a.a. downstream of the furin-recognition site were deleted, produced a 2- to 3-fold increase in plasma FVIII activity in vivo following AAV8-mediated delivery, with sustained expression for up to 20 weeks and no evidence of hepatotoxicity. Although the constructs showed only minimal differences in vitro, in vivo **ΔRHQR10** reached 50–200% of FVIII-N6’s activity after a 2 × 10^12^ vg/kg injection. The effect was particularly pronounced in male animals, in which activity levels were approximately threefold higher than in females [144].

The patent application (WO2024255448A1) describes **FVIII-L1**, the variant used in NGGT003 therapy, in which the furin cleavage site is replaced with the short, flexible peptide sequence GGGGS. FVIII-L1 demonstrated a pronounced hemostatic effect compared with FVIII-SQ. The data indicate a trend toward higher plasma FVIII antigen levels and activity at six weeks following AAV-mediated delivery. However, no statistically significant differences in either parameter were observed between FVIII-L1 and FVIII-SQ at doses of 8e11 and 4 × 10^12^ vg/kg in the mouse model [154].

#### 3.2.2. Preservation of Glycosylation Sites

Due to the fact that FVIII secretion depends on the mannose-binding transporter LMAN1, attempts were made to shorten FVIII while keeping the B-domain’s glycosylation sites intact. The linker variants retained 29–226 a.a. from the N-terminal B-domain region and were selected according to the number of potential N-linked glycosylation sites. Keeping 6 or 8 sites gave a 5- to 10-fold rise in secretion efficiency and reduced markers of ER stress by about threefold in mice after hydrodynamic injection, relative to WT and FVIII-BDD [125,155], whereas loss of most N-linked glycosylation sites markedly reduced FVIII secretion. The shortest effective variant, **FVIII-N6**, retained 6 motifs and 226 B-domain residues [125]. FVIII-N6 localized mainly to the Golgi apparatus, unlike FVIII-BDD [156]. Adenoviral vector (Ad) delivery of FVIII under the control of the phosphoenolpyruvate carboxykinase promoter resulted in long-term FVIII activity and phenotypic correction for up to 74 weeks, with low anti-FVIII antibody titers. In vitro, combining the FVIII-N6 design with the F309S substitution produced an additive improvement in secretion [125]. However, mice injected with a vector carrying both mutations together showed neither prolonged activity nor low anti-FVIII antibody titers observed in mice receiving N6-FVIII alone [156].

**Figure 5:**
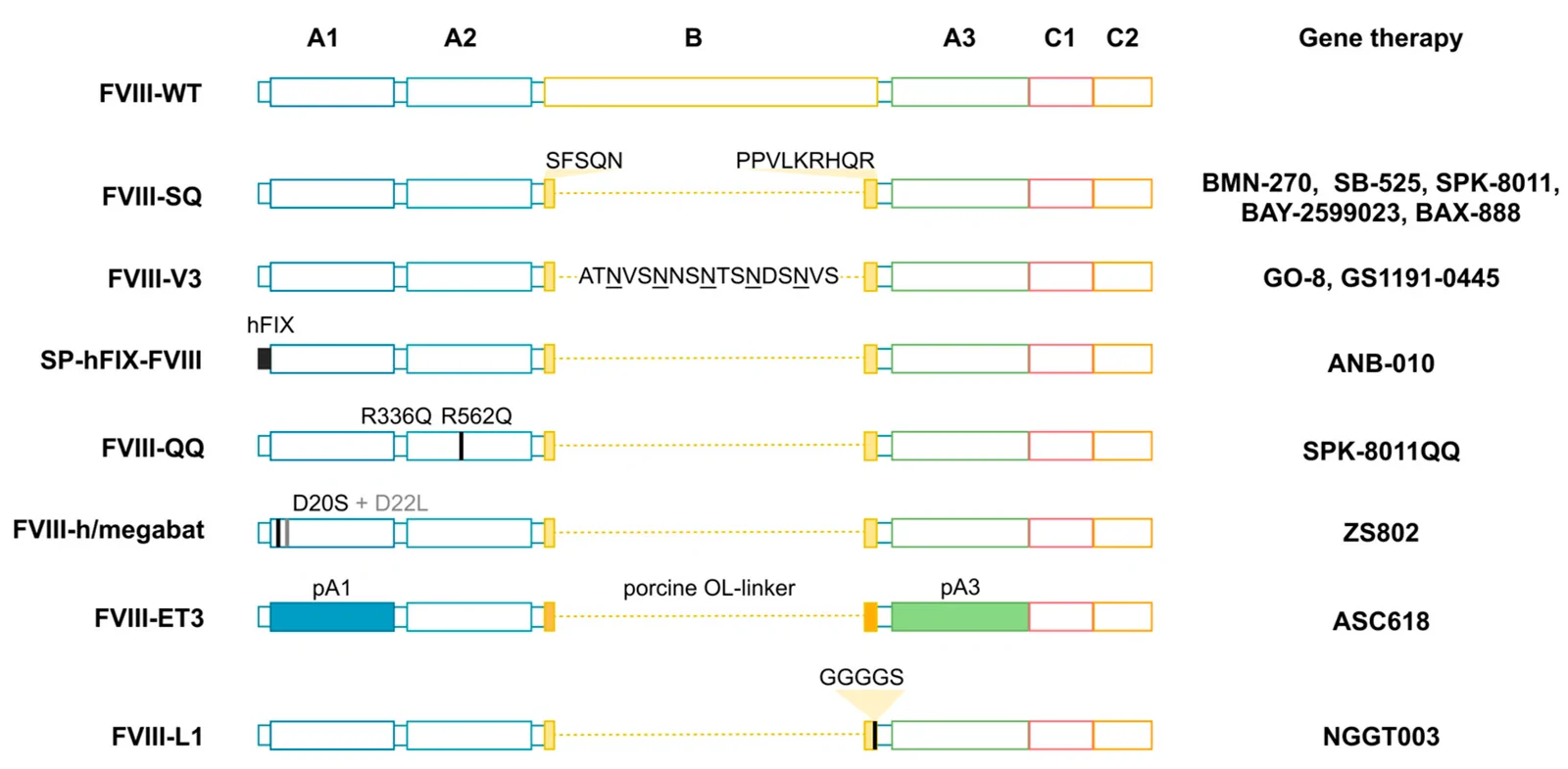
Engineered FVIII variants used in AAV-mediated gene therapy. FVIII-WT, FVIII-SQ [130], FVIII-V3 [132], SP-hFIX-FVIII [157], FVIII-QQ [158], FVIII-h/megabat [159], FVIII-ET3 [160]. Gray lettering indicates supposed substitutions. Filled regions indicate pFVIII-derived sequences. The OL linker corresponds to the native 24-a.a. pFVIII linker sequence. pA1 and pA3, porcine A1 and A3 domains. Underlined residues indicate N-glycosylation sites within the linker.

Despite this success in Ad-based gene therapy, FVIII-N6 proved incompatible with AAV delivery, since its cassette exceeded the capsid’s packaging capacity, producing heterogeneous viral particles from incompletely packaged DNA fragments. The resulting variant, hFVIII-V3, was designed instead to shrink the protein while keeping its N-glycosylation sites intact, to preserve effective FVIII secretion (Figure 5). Built on the FVIII-SQ backbone, it adds a 17-a.a. peptide carrying 6 glycosylation sites to the center of the SQ linker. Two ways of incorporating the consensus sequences were tested: a minimal-length peptide retaining just the 6 asparagine glycosylation triplets (NXT/S) from the B-domain (hFVIII-V3) and one retaining three native B-domain-flanking a.a. at each triplet (hFVIII-V1). Although both assembled into homogeneous, full-length vector genomes, only hFVIII-V3 showed a two-fold rise in functional plasma factor over FVIII-N6 in a mouse model after AAV delivery, highlighting the importance of how retained glycosylation sites are arranged within the linker. Notably, hFVIII-V3 also has a higher SC content (170 kDa) than hFVIII-SQ, despite retaining the furin recognition site [132].

### 3.3. Signal Peptide Modifications

The SP’s properties, hydrophobicity, length, and charge affect the rate of translocation, its coordination with the ribosome, and the efficiency of the subsequent folding environment. Proteins with a low-hydrophobicity SP are known to be translocated with the help of Sec63, which recruits and activates the ATPase BiP to drive the protein directly into the ER; this route can involve a pause in translocation, allowing partial folding of the growing chain in the cytosol and likely proteasomal degradation. Highly hydrophobic SPs, by contrast, do not require Sec63 or BiP at all [50]. The precise mechanics of FVIII translocation, and its specific molecular partners, have yet to be established, but attempts have been made to replace its SP with heterologous variants.

A set of synthetic peptides predicted using a hidden Markov model (HMM—a method used to identify and characterize a.a. motifs), together with the natural SPs of apolipoprotein C-III and alpha-1-antichymotrypsin (both with a high HMM bit score), gave lower or comparable FVIII secretion relative to the native SP. The HMM bit score reflects how closely a sequence matches the statistical SP model. These results indicate how important it is for the SP to suit FVIII’s particular biosynthetic requirements [161]. Zhao et al. tested the hypothesis that replacing it with the albumin SP, from a highly secreted, hepatocyte-specific protein, might boost FVIII secretion after AAV delivery but found no advantage from this strategy [144]. SP-hFIX proved the most effective choice for the ANB-010 therapy, giving secretion and activity levels more than double those of the native variant in vitro. The mouse α-lactalbumin SP also outperformed the native SP, though it fell short of SP-hFIX (Figure 5) [157].

### 3.4. UPR and the FVIII Variant Designed to Overcome It

FVIII remains one of the most challenging proteins to express recombinantly, as its production in both in vitro and in vivo systems is substantially lower than that of other coagulation factors [58]. Under comparable conditions, its homologous protein FV is secreted approximately 5–10 times more efficiently [162,163]. Experimental data indicate that FVIII’s low production yield cannot be overcome merely by increasing the expression burden. For example, B-domain deletion can markedly increase FVIII mRNA abundance and primary translation, while yielding only a modest increase in secreted protein because of intracellular retention and inefficient secretion [71]. Moreover, energy depletion and inhibition of oxidative phosphorylation selectively impair the anterograde trafficking of FVIII through the early secretory pathway, without affecting FV or von Willebrand factor (vWF) in the same cells [59]. These features make FVIII a challenging candidate for AAV-mediated gene therapy, as high expression levels can lead to intracellular aggregation and limited secretion of functional protein.

Early studies found that high expression of the chaperone BiP correlates with reduced secretion of human FVIII (hFVIII) [59,164]. This led to the view that BiP binding contributes to the formation of aggregates, which involve approximately one-third of all newly synthesized FVIII molecules. Accordingly, efforts were made to identify and remove the sequence elements responsible for BiP binding [59,165]. Expression of an FVIII variant lacking the A1 domain increased approximately 10-fold compared to wild-type FVIII. The responsible region was further localized to the 110 C-terminal a.a. of the A1 domain, and replacing this segment with the homologous sequence from FV likewise increased secretion by approximately 10-fold [166]. In parallel, FVIII secretion was found to be limited at an ATP-dependent transport step: ATP depletion promotes aggregate formation, whereas normal ATP levels facilitate their dissociation within the ER lumen. Notably, FVIII was the first known protein shown to form aggregates in the ER independently of disulfide-bond formation [165].

Current evidence has led to a reassessment of BiP’s role in FVIII biosynthesis. BiP was found to be part of an ATP-dependent system that prevents protein aggregation. A functional deficiency of BiP relative to FVIII during increased synthesis of FVIII and other proteins or reduced glucose metabolism leads to the accumulation of aggregated FVIII in the ER [58].

However, BiP preferentially binds low-molecular-weight FVIII aggregates and participates in the UPR, a conserved mechanism that restores cellular homeostasis upon the accumulation of misfolded proteins. Activation of the UPR increases chaperone expression to improve protein folding. However, it can also negatively affect the efficiency of gene therapy by reversibly inhibiting protein biosynthesis and activating the endoplasmic-reticulum-associated degradation (ERAD) pathway. Moreover, prolonged UPR activation can lead to apoptosis. The UPR is mediated through three signaling pathways (PERK, ATF6, and IRE1α), which bind BiP in the ER lumen. When chaperone demand is high, BiP dissociates from these membrane-associated partners and binds FVIII, thereby activating downstream cytoplasmic signaling cascades. IRE1α forms a complex with the Sec61 translocon to degrade specific mRNA substrates through regulated IRE1-dependent decay (RIDD), thereby reducing the protein-folding burden on the ER [162,167].

FVIII aggregates were shown to have an amyloid-like architecture, forming fibrils approximately 5 nm in diameter within the ER lumen. Their polymerization is initiated by antiparallel β-sheet assembly, nucleated by a 79-a.a. segment termed Aggron (residues 253–331) in the A1 domain [59]. FVIII aggregation can be reversible: restoration of glucose metabolism and ATP-dependent BiP activity promotes disaggregation, refolding, and subsequent secretion of functional FVIII, likely rescuing approximately half of the aggregated protein [58]. Blocking either autophagy or proteasome-dependent degradation does not increase FVIII aggregation after glucose replenishment, indicating that these pathways make only a minor contribution to the clearance of FVIII aggregates [59].

Considering that protein aggregation is concentration-dependent, FVIII likely has an expression threshold above which intracellular aggregation, ER stress, and the risk of death of transduced cells increase. In 2024, it was first shown that the durability of FVIII expression after AAV-mediated gene delivery depends on the plasma FVIII antigen concentration achieved. At elevated plasma FVIII levels (>1.5–3 nM), expression declined substantially over time compared with the low- and normal-expression groups. This decline was accompanied by a reduction in liver vector copy number, with no evidence of transcriptional silencing, consistent with the hypothesis that transduced cells are lost over time. The proposed mechanism is activation of the pro-apoptotic branch of the UPR, as increased expression of the pro-apoptotic marker CHOP was observed at week 8 in the high-FVIII-expression group. However, by week 72, elevated CHOP levels were detected in all groups, including the control group. These findings therefore support, but do not establish, a direct causal relationship between high FVIII expression, UPR activation, and cell death [158].

It remains unknown whether the cellular stress response to FVIII differs between hepatocyte-based models used for in vitro testing and AAV-mediated delivery to LSECs, the endogenous site of FVIII synthesis. This distinction is particularly important in gene-therapy settings, where transgene expression levels may be artificially elevated.

Alternatively, stress and inflammation may suppress FVIII translation without loss of transduced hepatocytes. In mice, FVIII production declined despite persistent vector DNA and mRNA, while IL-15 blockade partially preserved expression. Thus, expression durability may depend on per-cell FVIII production and the interplay between cellular stress and immune responses [168].

***Overcoming intracellular retention of FVIII***. Optimizing FVIII for more efficient intracellular transport has been a goal of its engineering since the start and remains one today. Two broad approaches can be distinguished: early rational design based on studies of its structure and trafficking, and a newer approach that applies favorable orthologue mutations to human FVIII.

Early studies identified a region of the A1 domain thought to interact with BiP [169], and, on the theory that this chaperone interaction was impeding secretion, the substitution F309S, borrowed from the homologous FV, was introduced into FVIII. F309S doubled the secretion of functional FVIII and reduced the dependence of secretion efficiency on cellular ATP levels, while **FVIII-F309S** itself retained wild-type coagulant activity, thermal stability, and thrombin activation [145].

Current understanding holds that colocalization with BiP actually results from amyloid formation by regions of the A1 domain, and that F309S reduces ER retention by adding a polar serine to the A1 domain’s hydrophobic pocket, which prevents these amyloid structures from forming. The same change also makes the protein 10-fold more sensitive to chelating agents by disrupting Cu^+^ coordination at residue C310, in other words, weakening the affinity between the light and heavy chains [59]. Searching for further substitutions in the hydrophobic pocket next to F309S turned up one synergistic mutation, **L303E**, which enhanced F309S’s effect 1.5-fold, though it was never applied further [145].

### 3.5. Variants of FVIII with Increased Stability

#### 3.5.1. Resistance to Inactivation

Once activated by thrombin, FVIIIa is rapidly inactivated as its heterotrimeric structure loses stability through spontaneous dissociation of the A2 domain and cleavage by APC (at R336, R562) and thrombin.

The variant **FVIII-IR8** (del795–1689/R336I/R562K/R740A), with the B-domain deleted, was built by sequentially eliminating the thrombin- and APC-cleavage sites through missense mutations, so that the A2 domain stays covalently bound to the light chain even after FVIII-IR8 activation [133]. The protein retained 38% of its peak activity in vitro after 4 h, compared with hFVIII-WT (inactivated by thrombin within 10 min). FVIII-IR8 showed fivefold higher specific activity than FVIII-WT, an order-of-magnitude lower affinity for vWF (consistent with the deleted acidic region of the LC), and resistance to inactivation by the monoclonal antibody ESH8, which is thought to stabilize the FVIII-vWF interaction. Another observation was that a protein variant with a short linker between the A2 domain and the light chain lost activity, underscoring the importance of optimizing linker length. In FVIII-IR8, residues 741–794 of the B domain serve as the linker [133,170]. Despite these promising early results, sustained coagulant activity from FVIII-IR8 could not be confirmed in a mouse model using ex vivo lentiviral vector (LV) transduction and platelet expression of IR8 [171].

Across both clinical and preclinical settings, high peak FVIII antigen levels appear to be a major determinant of declining transgene expression. This highlights the need to achieve equivalent hemostatic activity at lower FVIII antigen concentrations [158]. This strategy was implemented in the FVIII-QQ variant, which carries the R336Q and R562Q substitutions at two sites of proteolytic inactivation by activated protein C (APC). FVIII-QQ showed a reduced rate of APC-mediated inactivation, providing a 30% activity (CSA) increase in APC-sensitive ex vivo assays, without changes in either the A2-domain dissociation rate or specific activity [158,172]. In vivo, these mutations reduced blood loss and achieved normal FVIII activity at antigen concentrations 3–5-fold lower than those required for hFVIII-BDD. In an immunocompetent mouse model, FVIII-QQ did not increase the risk of a humoral immune response, as assessed by the Bethesda inhibitor assay and anti-FVIII IgG titers [158].

#### 3.5.2. Enhancement of Interdomain Interaction Interfaces

An alternative strategy is to directly tether the A2 domain to the A3 domain through a covalent linkage. To achieve this, cysteine pairs C664/C1826 or C662/C1828 were introduced into FVIII-BDD, creating an interdomain A2-A3 disulfide bond. Formation of this bridge prevents A2-domain dissociation, one of the principal mechanisms of spontaneous FVIIIa inactivation [173,174]. The resulting variants, referred to in the literature as **FVIII-CC**, exhibited an approximately fivefold slower rate of activity loss than FVIII-WT and were more stable than FVIII-IR8, despite lacking mutations that eliminate APC recognition sites [146]. The authors attributed this to increased affinity for von Willebrand factor (vWF), which prolongs FVIII’s plasma half-life, or to the possibility that the A2 domain in IR8-FVIII can still dissociate within the range permitted by its flexible linker [174]. Accordingly, engineering IR8 variants with a more conformationally constrained linker may be a promising direction. It should be noted, however, that the functional effects depend on the position of the introduced disulfide bridge (unlike hFVIII-BDD-C664/C1826, the hFVIII-BDD-C662/C1828 mutant retained the specific activity of wild-type FVIII-BDD) [173].

The half-life of FVIII may be extended by strengthening hydrophobic interactions at the A1-A2 and A2-A3 domain interfaces. Substitution of the charged residues D519, E665, and E1984 with alanine or valine increased the stability of both FVIII and FVIIIa while maintaining activity comparable to that of hFVIII-BDD. These variants also showed greater resistance to thermal and chemical denaturation, along with reduced rates of FVIII and FVIIIa decay [175,176]. The most effective substitutions targeted Glu1984, which retained more than 90% activity 40 min after activation and showed a twofold reduction in the rate of thermal inactivation [175]. At position E665, Val, with its bulkier hydrophobic side chain, was more effective than Ala. All mutations reduced the apparent Kd of the A2 domain for the WT A1/A3C1C2 dimer, indicating enhanced A2-domain affinity for the remaining FVIIIa subunits [177]. They did not affect the amount of protein secreted in vitro and did not prevent APC-mediated inactivation through cleavage at Arg336 and Arg562 [175].

Some combinations of these mutations (Asp519Ala/Glu665Ala, Asp519Val/Glu665Val, and Asp519Val/Glu1984Val) were referred to as **FVIII-VV** [113]. These combinations achieved a 7–10-fold reduction in the rate of FVIIIa decay relative to hFVIII-BDD and an approximately 1.5–3-fold reduction relative to the best single mutation. Interestingly, combining hydrophobic substitutions at positions 665 and 1984 attenuated the beneficial effects of the individual mutations, likely because these residues are located within the same structural cluster [176].

Thus, strengthening hydrophobic interfaces involving the A2 domain may be an effective strategy for engineering FVIII variants with an extended functional half-life. However, hydrophobic substitutions must be designed with steric precision, as both differences in side-chain structure and replacement of neighboring polar residues at interaction interfaces may instead reduce protein stability [175,177,178].

Attempts were made to further improve the FVIII-D519V/E665V variant by introducing multiple additional mutations, including Ala108Ile in the hydrophobic pocket at the A1-C2 interface, and substitution of the residues flanking Arg336 (residues 333–339 PQLRMKN) with the sequence flanking the more slowly cleaved Arg562 site (residues 559–565 VDQRGNQ). The latter modification is designated FVIII-336(P4–P3′)562. When combined with D519V/E665V, **FVIII-336(P4–P3′)562** showed a 14-fold lower rate of FVIIIa decay than FVIII-D519V/E665V alone [176].

More recently, a triple FVIII variant was engineered on the FVIII-D519V/E665V background by introducing the additional K1813A substitution in the FIXa-binding region of the A3 domain. This mutation further increased hemostatic activity in both in vitro and in vivo models, presumably by promoting formation of a more stable FVIIIa–FIXa complex and reducing the rate of A2-domain dissociation [179]. In the tail-clip assay following systemic protein administration, the D519V/E665V/K1813A triple mutant exhibited an approximately eightfold greater hemostatic potential than FVIII-WT and a fourfold greater potential than D519V/E665V. In vitro, D519V/E665V/K1813A outperformed both FVIII-WT and D519V/E665V across several measures: its specific activity in the one-stage clotting assay (OSA) was approximately 3.5-fold higher than that of FVIII-WT, FXa generation was approximately 2.3-fold greater, and the apparent Kd for FIXa was approximately 1.4-fold lower. The triple mutant also showed an approximately 50-fold slower rate of FVIIIa inactivation in the one-stage assay, an approximately eightfold greater thrombin-generation potential, and improved rotational thromboelastometry parameters [147].

#### 3.5.3. Reducing Affinity for Clearance Receptors

Although reducing LRP1-mediated clearance is an attractive strategy for prolonging FVIII circulation following AAV-mediated gene therapy, a substantial proportion of FVIII removed from the bloodstream has already been inactivated. Therefore, extending its plasma half-life does not necessarily translate into increased functional activity.

In the context of engineering FVIII variants with an extended half-life, selective substitution of lysine residues involved in binding to cluster IV of LRP1 has been proposed [122]. However, the LRP1-binding site overlaps with the binding interfaces for vWF and phospholipids, creating a risk that mutations could compromise FVIII stability or membrane binding. Because such selective variants have not yet been developed or tested, this concept remains hypothetical. Nevertheless, the growing body of structural data on FVIII complexes with inhibitory antibodies provides a foundation for the rational design of new FVIII variants [180,181].

### 3.6. Disulfide-Bond Engineering

A negative-selection strategy based on substitutions at cysteine residues showed that, among the eight disulfide bonds present in FVIII-WT, only the Cys1899–Cys1903 bond was dispensable. Indeed, this disulfide bond appeared to impair proper intracellular folding and trafficking of FVIII. Structural analyses indicated that this cysteine pair is unique in being surface-exposed within the A3 domain, whereas the other seven disulfide bonds are buried in the hydrophobic core of the protein. Moreover, the corresponding disulfide bond is absent from factor V (FV), an FVIII homolog that is secreted at levels approximately 10–20-fold higher.

The engineered double mutants, C1899S/C1903S and C1899G/C1903G, showed on average 1.2- to 1.6-fold higher secretion than FVIII-WT, consistent with the effect observed when these mutations were introduced into FVIII-BDD in vitro. C1899G/C1903G was added to the FVIII-N6 variant to generate FVIII-N6-DM, increasing FVIII secretion 2.2-fold without altering mRNA levels. The additional incorporation of the previously described F309S point mutation further increased the level of secreted protein by 3.2-fold, generating FVIII-N6-DM-F309S. Collectively, these modifications resulted in a 35- to 45-fold increase in secretion of FVIII-N6-DM-F309S relative to FVIII-WT in CHO cells [148].

### 3.7. Ortholog-Based Design

Orthologous a.a. substitutions have historically been a major source of ideas for optimizing the hFVIII sequence. This approach involves comparing hFVIII with naturally occurring FVIII orthologs (primarily from mammals) that differ in secretion, activity, and stability, then introducing selected substitutions into hFVIII, and identifying variants associated with improved FVIII function. However, the molecular roles of these a.a. substitutions (including their effects on protein folding, interaction with BiP, export from the ER, FVIIIa stability, and Xase function) are typically established only after functional testing. Thus, the study of orthologs enables the development of novel enhanced FVIII variants through both screening-based empirical and rational approaches.

Some FVIII orthologs do appear to offer potential therapeutic advantages over human FVIII. The most compelling examples are porcine FVIII (pFVIII), which is secreted substantially more efficiently than human FVIII by mammalian cells despite comparable mRNA levels, and canine FVIII (cFVIII), which exhibits higher specific activity and greater stability of its activated form [140,182,183,184]. In contrast, murine and ovine FVIII have not demonstrated a consistent biosynthetic advantage over hFVIII across experimental systems [140,151,185], although both have been reported to exhibit slower A2-domain dissociation following activation [140,149,150,185]. Nevertheless, these sequences remain valuable for comparative analysis by comparing FVIII orthologs to identify conserved and variable positions associated with FVIII secretion, stability, activity, and immunological properties. Numerous hFVIII variants for therapeutic applications have been developed on the basis of such an approach.

The porcine ortholog, pFVIII-BDD, differs from hFVIII-BDD at 228 a.a. positions and retains approximately 83% sequence identity outside the B domain [149,182,183]. Its shortened linker between the heavy and light chains, termed the OL linker in FVIII-OL, differs from the SQ linker in both sequence composition and length. At comparable mRNA levels, pFVIII secretion by mammalian cells is 5- to 16-fold higher than that of hFVIII-SQ and mFVIII-BDD, depending on the cell line and the assay used to quantify FVIII activity (OSA or CSA) [149,160,182,183,184]. Despite similar thermal stability (Tm 56 °C for pFVIII-BDD and 58 °C for hFVIII-SQ), the superior biosynthetic performance of pFVIII has been associated, at least in part, with weaker induction of UPR markers, by approximately 1.2- to 2.6-fold [183]. In Japan, recombinant pFVIII (Susoctocog alfa, Obizur) was approved in 2024 for the control of bleeding episodes in adults with acquired HA—a condition caused by inhibitory antibodies against human FVIII [186].

The chimeric variant **FVIII-ET3**, originally designated HP47, was generated by introducing selected porcine FVIII sequences from the A1 and acidic a3 regions into the hFVIII-BDD backbone [160,187]. These domains were sufficient to confer a secretion efficiency comparable to that of pFVIII. FVIII-ET3 contains 149 porcine a.a. residues, corresponding to approximately 11% of its sequence, while retaining 89% sequence identity with hFVIII-BDD [139]. The 24-a.a. porcine OL linker used in this construct does not confer an additional expression advantage over the SQ linker [187].

Depending on the experimental system, FVIII-ET3 exhibited a 1.5- to 3-fold increase in expression after transient transfection in COS1 and BHK-M cells and up to a 10- to 100-fold increase in other models. This enhancement has been attributed, at least in part, to 13 porcine substitutions in the A1 region spanning residues 226–336, a region implicated in BiP interaction, and potentially to differences in N-glycosylation sites that could influence trafficking through the ER and GA [125,187].

**ET-801i** is a further optimization of FVIII-ET3 that additionally incorporates porcine C1-domain sequences and three Ala substitutions in the A2 domain. It retains 88% a.a. sequence homology with FVIII-SQ. When expressed using an LV, ET-801i production in vitro was reported to be 16- to 160-fold higher than that of hFVIII-BDD. However, the precise positions of the a.a. substitutions responsible for this effect were not disclosed by the authors. Consequently, the molecular basis for the enhanced expression remains uncertain [124].

The development of the **FVIII-X5** variant, which retains secretion efficiency comparable to that of pFVIII, involved iterative narrowing of the regions containing a.a. differences between FVIII orthologs within the heavy chain of hFVIII [137]. Initially, a panel of FVIII-HC variants was generated and tested in vitro. These variants contained porcine replacements of segments of varying length within the A1 and A2 domains and the acidic a1 linker and were co-transfected with the human FVIII-LC. In the most successful chimera, which contained the pFVIII sequence spanning residues 82–176 within the A1 domain, ten substitutions were identified. Their individual contributions were assessed by negative selection. This ultimately yielded the B-domain-deleted FVIII-X5 variant, which carries five key porcine substitutions (I86V, A108S, G132K, M147T, and L152P). These substitutions were necessary for enhanced secretion, producing an approximately 2.5-fold increase relative to hFVIII-BDD, and did not confer an additional immunogenicity risk compared with hFVIII-BDD.

The authors also noted that the SC form appeared as a more prominent band for FVIII-X5 than for FVIII-BDD in the experimental data, although this observation was not quantified. In in vivo studies using an AAV8 vector driven by the TTR promoter, FVIII-X5 produced more than a tenfold increase in FVIII activity and sustained high plasma FVIII levels for 22 weeks after administration at doses of 1 × 10^11^–4 × 10^11^ vector genomes per mouse. The outcome was further substantially improved by codon optimization [137].

Canine B-domain-deleted FVIII (cFVIII-BDD) is of interest as an ortholog with approximately 3- to 7-fold higher specific activity than hFVIII-SQ. Its activated form, cFVIIIa, has an approximately threefold longer functional half-life, owing to increased stability of the A2 domain [140,188]. cFVIII-BDD itself has been reported to display roughly threefold higher specific activity than human FVIII-BDD. Combining the human heavy chain with the canine light chain increased coagulation activity by approximately 18-fold [136]. Therefore, cFVIII has primarily served as a template for engineering variants with enhanced coagulant activity and improved FVIIIa stability rather than for improving secretion.

The hybrid variant **FVIII-JF12** was designed by an approach analogous to that used for FVIII-X5, namely by identifying functionally important a.a. residues in the light chain of canine FVIII (cLC) [188]. At the first stage of selection, 33 FVIII variants were tested. These variants were generated by combining regions of cFVIII with hFVIII-BDD within the hLC domains. The cFVIII region spanning residues 1857–2147, which encompasses parts of the A3-C1 domains, was identified as positively correlated with increased factor activity. At the next stage, 26 single substitutions were evaluated using in vitro negative selection—ten of them resulted in increased FVIII activity. Based on these results, a human FVIII-BDD variant containing 10 a.a. substitutions corresponding to residues in the LC of canine FVIII was developed as **FVIII-JF10** with activity comparable to that of cFVIII. Interestingly, during the selection process, the authors identified a clone with activity exceeding that of cFVIII. Further characterization revealed two additional spontaneous a.a. substitutions, S2157N and R2159H, which were absent from both the human and canine variants. These substitutions did not confer a functional advantage in the absence of the ten previously identified canine substitutions.

In vivo testing of the newly identified **FVIII-JF12** variant, carrying 12 substitutions (V1857I, H1859R, M1907K, M1926K, L1975V, A1993V, H2007Q, E2066D, K2085M, Q2113H, S2157N, and R2159H), using both single- and dual-chain AAV8 vectors for transgene delivery, confirmed its higher coagulation activity relative to hFVIII-SQ, without reducing secretion efficiency. The new variant reduced the Km and increased the Vmax of the Xase complex; however, the structural basis for its enhanced performance and the effect of the additional substitutions on inhibitor formation remain to be investigated [188].

**h/megabat FVIII** (ZS802). Using ortholog-based engineering and structural modeling, Dong et al. identified the N-terminal D1 region (residues 1–187) of megabat FVIII as contributing to enhanced FVIII activity. D20S or D20S/G22 megabat substitutions increased FVIII coagulant activity in HEK293 culture supernatants by 2.5–3.5-fold relative to FVIII-SQ. One selected variant was incorporated into the AAV gene-therapy cassette ZS802, which is currently being evaluated in clinical trials at doses ranging from 2 × 10^13^ to 6 × 10^13^ vg/kg [159].

### 3.8. Gain-of-Function Variants

**FVIII-SQ (Ver.4)** is an engineered FVIII variant developed through the identification of conserved orthologous substitutions [149]. The authors sequentially compared hFVIII-SQ with porcine and cFVIII and selected a.a. differences shared between these species that involved altered physicochemical properties and then retained positions that were also conserved in ovine and bovine FVIII. The resulting construct contained 36 a.a. substitutions distributed across all FVIII domains. These changes accounted for 2.5% of the total protein sequence and introduced new putative glycosylation motifs: an N-linked glycosylation motif at N213 and potential O-linked glycosylation sites at T1653 and T1654.

Following AAV8-mediated delivery in mice, FVIII-SQ (Ver.4) yielded a 4-fold higher FVIII:Ag level than hFVIII-SQ. This was accompanied by an 8.2-fold increase in FVIII activity in the OSA and a 3.4-fold increase in the CSA, despite comparable or lower vector genome load and hepatic mRNA expression. In Huh7 cells, FVIII-SQ (Ver.4) reduced intracellular retention of the LC and attenuated IRE1/XBP1-dependent UPR signaling, consistent with more efficient secretion. Purified FVIII-SQ (Ver.4) showed 3.6-fold and 1.5-fold higher specific activity in the OSA and CSA, respectively. Its affinity for FIXa was also increased, as indicated by a decrease in Kd from 1.3 to 0.57 nM. This improvement may result from structural rearrangement at the FVIII–FIXa interface, particularly involving the I566M substitution. According to predicted structures, in the I566M variant, the adjacent M567 residue is oriented toward FIXa. This conformation is proposed to displace an α-helix of FIXa, creating space for FIXa R338 to form a hydrogen bond with FVIII Y636. In addition, M566 is positioned near FIXa T343 and may likewise contribute to hydrogen-bond formation [149].

However, the M539L substitution destabilized FVIIIa by accelerating A2-domain dissociation 2.6-fold, likely owing to disruption of a methionine–aromatic interaction motif at the A1-A2 interface. Despite enhanced FXa generation, thrombin-generation potential remained comparable to that of hFVIII-SQ [149].

In macaques, AAV5 and AAV8 vectors carrying a codon-optimized, CpG-depleted expression cassette produced supraphysiological FVIII activity at doses from 2 × 10^12^ to 6 × 10^12^ vg/kg. However, FVIII activity subsequently declined in 6 of 7 animals because of the development of nAbs against human FVIII. Thus, FVIII-SQ (Ver.4) combines improved secretion with enhanced functional activity, whereas its long-term immunological compatibility remains a key limitation [149].

The phylogenetic approach of ancestral sequence reconstruction to FVIII design is based on the premise that extant orthologs have been shaped by natural selection to maintain hemostatic balance in each species rather than to maximize FVIII activity. Specifically, the authors hypothesized that reduced FVIIIa activity or stability may have emerged in the hominid lineage as protection against thrombotic risk; however, this interpretation remains an evolutionary hypothesis.

A phylogenetic tree was constructed from 47 mammalian FVIII sequences, and putative ancestral sequences were reconstructed. Among 14 synthesized variants, **An-53**, an inferred common ancestor of primates and rodents that retained 95% sequence identity with hFVIII, demonstrated a 12-fold increase in in vitro production. Following administration of AAV8-FVIII-An-53 to mice at a dose of 2 × 10^11^ vg/kg, FVIII activity exceeded 0.45 IU/mL at weeks 2–4 and remained higher than that of hFVIII even at a 10-fold lower dose. The molecular basis of the enhanced expression of ancestral FVIII variants has not been established. Potential contributing factors include increased translational efficiency, improved folding or secretion, and reduced activation of the UPR [150].

Mashausi et al. reported that introduction of the single **P290T** substitution increased the specific coagulation activity of FVIII in vitro and reduced blood loss in an FVIII-deficient mouse tail-clip assay following hydrodynamic plasmid delivery. However, the molecular mechanism underlying this effect has not been established [151].

Preliminary findings presented at the 2023 ASH Annual Meeting described evolution-directed gene engineering (EDGE) as an approach for identifying gain-of-function FVIII variants. **K659M/V** showed approximately six-fold higher specific activity and restored hemostasis at lower protein concentrations in HA mice, while **K659C** increased plasma FVIII activity following AAV8-mediated delivery [135].

In 2009, FIX Padua was described as a naturally occurring gain-of-function FIX-R338L variant identified in patients with X-linked thrombophilia and exhibiting 5–10-fold higher specific activity [189]. Its success in AAV-mediated gene therapy for HB has made the search for an analogous hyperactive FVIII variant particularly compelling. In 2024, the first FIX Padua analogue for HA, **FVIII-Aurora** (R571S), was reported as the first naturally occurring gain-of-function FVIII variant [190]. It was identified in an 18-year-old patient with recurrent life-threatening arterial and venous thromboses beginning in the perinatal period. FVIII activity ranged from 300% to 900% despite normal FVIII antigen levels. Recombinant Aurora-FVIII exhibited an approximately 6-fold increase in activity in the one-stage assay while showing near-normal activity in the chromogenic assay [191]. A recent study attributed this hyperactivity to a 10–20-fold increase in the affinity of FVIIIa for FIXa [152]. In addition, formation of a stable intrinsic Xase complex sterically shields FVIIIa from APC-mediated inactivation and reduces A2-domain dissociation. The study presents a structural model showing the close spatial proximity of R571S to the APC cleavage site at R562. In mouse models, FVIII-Aurora provided 4–5-fold greater hemostatic efficacy and induced a pronounced prothrombotic phenotype. Although this variant has not yet been used therapeutically, it may serve as a basis for AAV constructs capable of achieving hemostasis at lower FVIII concentrations and vector doses, provided that thrombogenicity is rigorously controlled.

### 3.9. Overcoming Immunogenicity in AAV-Mediated Gene Therapies

FVIII replacement therapy has limited efficacy in a subset of patients, requires repeated infusions, and is associated with inhibitor development in approximately 30% of patients with severe HA [192]. Inhibitory antibodies against FVIII may arise because null *F8* mutations result in the complete absence of FVIII protein, thereby preventing the establishment of central immune tolerance to FVIII during immune system development. Consequently, exogenously administered FVIII may be recognized as a foreign antigen [193]. This is a serious complication associated with increased morbidity and mortality [194]. While immune tolerance induction (ITI protocol) with regular FVIII infusions can eradicate inhibitors, it fails in about one-third of patients [195].

Inhibitor development is a complex, multifactorial, and actively investigated process. Risk factors for inhibitor development include genetic factors, such as *F8* mutations and polymorphisms in genes encoding human leukocyte antigen (HLA) molecules, CTLA-4, and cytokines; epigenetic immunological factors (including Treg function, the cytokine milieu, and Th2/Tfh responses); treatment-related factors (timing and dose of initial FVIII exposure); and environmental factors [196,197].

It has long been recognized that inhibitors develop more frequently in patients with severe HA, occurring in up to 25–30% of cases, than in those with severe HB, in whom the incidence is 2–5%. In mild HB, inhibitor antibodies may be entirely absent [2,198]. Traditionally, this difference has been attributed to the higher prevalence of missense mutations in HB than in HA, affecting *F9* and *F8*, respectively. Such mutations often result in the production of dysfunctional, but still detectable FIX protein. In addition, FIX is structurally homologous to other vitamin K-dependent coagulation factors. Together, these factors may enable partial establishment of central and peripheral immune tolerance to FIX, a process that is less likely to occur for FVIII. The smaller size of FIX relative to FVIII may also facilitate transplacental passage of FIX and contribute to the development of pregnancy-associated immune tolerance. Recent single-cell RNA sequencing of PBMCs from patients with severe HA or HB receiving replacement therapy revealed differences in immune-cell subset composition [199]. A higher proportion of B cells and a greater activation state were observed in the HA group, whereas the HB group showed a higher proportion of T cells, including more active CD4+ helper T cells.

However, human immune responses to clotting factors with distinct structural characteristics remain insufficiently characterized. *F8* gene therapy may potentially induce immune tolerance to the transgene product, as has been demonstrated for liver-directed gene therapies. This approach is supposed to overcome inhibitor formation problems in patients with HA [200,201,202,203,204].

Most existing FVIII-engineering approaches that have been successfully applied in replacement therapy (including PEGylation, fusion to the Fc fragment of IgG, and addition of polypeptide sequences to extend circulatory half-life) are not readily compatible with AAV-mediated therapy because of the limited packaging capacity of AAV vectors. In addition, it is not possible to selectively modify the metabolic pathways of producer cells in a manner that affects only the newly synthesized FVIII molecule. Therefore, the FVIII-SQ modifications for reduced immunogenicity discussed below are predominantly limited to individual a.a. substitutions.

***FVIII with reduced immunogenicity***. Rational immunological design of FVIII may involve eliminating specific glycosylated epitopes that contribute to T-cell recognition and inhibitor formation. To this end, the N2118Q substitution was introduced to eliminate high-mannose N-glycosylation in the C1 domain and a potential glycopeptide T-cell epitope. Following administration of AAV vectors encoding FVIII-BDD or **FVIII-N2118Q** to hemophilic mice at a dose of 1 × 10^12^ vg/mouse, FVIII activity in both groups reached approximately 70% of normal by week 4. However, after six subsequent weekly administrations of recombinant FVIII, inhibitors were detected in 100% of mice receiving FVIII-BDD and in 33% of those receiving N2118Q. By week 15, FVIII activity increased in both groups after a prior decline, which the authors interpreted as a possible indication of tolerance induction to the transgene product [153].

**FVIII-19M** is a variant designed to reduce immunogenicity through in silico identification of potential T-cell epitopes and the introduction of 19 orthologous substitutions outside regions critical for FVIII function, thereby reducing the likelihood of MHC class II presentation. The final construct contains the following substitutions: N79S, S112T, L160S, L171Q, V184A, N233D, I265T, N299D, Y426H, S507E, F555H, N616E, L706N, Y748S, K1837E, N2038D, S2077G, S2315T, and V2333A. In a dendritic cell-CD4+ T-cell co-culture system, the variant elicited lower helper T-cell proliferation than unmodified FVIII, suggesting that it may reduce the risk of inhibitory antibody formation. However, T-cell reactivity persisted in a subset of donors; therefore, these results support reduced, rather than eliminated, immunogenicity [134].

## 4. Hemophilia A Gene Therapy

### 4.1. First Attempts at Hemophilia A Gene Therapy

The earliest HA gene-therapy trials, using retroviral (RV) and adenoviral vector (Ad), came across two main problems: low FVIII antigen levels and consequent low activity after in vivo RV delivery to adult animals [205,206,207]; and extremely rapid loss of FVIII expression caused by immune-mediated hepatotoxicity and nAbs formation with Ad vectors [202]. Treating newborn animals proved a much more effective use of in vivo RV delivery. RV delivery of canine FVIII (cFVIII) to newborn mice fully corrected HA, with no inhibitors to the neoantigen detected by month 15, whereas adult animals showed rapid loss of cFVIII activity due to inhibitor formation. Canine neonates treated the same way gave similar results [208]. This likely reflects the antigen being presented early, together with expression localized to hepatocytes through use of a liver-specific promoter. However, concerns arise regarding the safety of using LV in vivo due to the increased risk of carcinogenesis and other defects related to RV integration, primarily in decondensed areas of chromatin associated with active transcription [209]. A Phase I trial delivering FVIII-BDD via RV in vivo proved unsuccessful with insufficient and unstable FVIII activity, leaving all patients to continue prophylactic treatment with concentrates [206].

Ad, however, carries high immunogenicity, including a heightened risk of immune responses to alloantigens that manifest as rapid loss of FVIII activity. Liver-specific promoters slowed this loss but did not solve the underlying problem [210]. In their study, Connelly et al. delivered cFVIII to dogs by Ad. Their subjects experienced sharp transaminase elevations after peak plasma cFVIII activity. This observation was attributed to an Ad-associated immune response and/or cFVIII overexpression. Moreover, FVIII inhibitor development was transient in 2 out of 4 dogs; however, it was significantly decreased by day 69. Additionally, purified FVIII injections at weeks 20 and 26 did not trigger repeat antibody formation in animals that previously formed inhibitory antibodies, although FVIII injection is usually accompanied by an anamnestic response. The authors suggested that the main reason for antibody development was the hepatotoxicity of Ad and that a less immunogenic vector would lower the frequency of anti-FVIII antibody formation [211]. This hypothesis is consistent with stable 5-month-long hFVIII expression in mice when using a lower viral load coupled with a liver-specific promoter [212]. The singular clinical trial of HA gene therapy using an Ad vector was terminated following the development of grade 3 hepatotoxicity in a single patient [213].

### 4.2. Ex Vivo Hemophilia A Gene Therapy with HSC Transplantation

Some success has also come from bone marrow transplantation using an oncoretroviral vector, though this approach demands extreme conditioning procedures to achieve successful engraftment [214]. Pioneering work demonstrated that targeting FVIII expression to platelets using the platelet-specific αIIb promoter (2bF8) restores hemostasis in HA mice with high-titer inhibitors. Efficacy was transferable via bone marrow (BM) transplantation or platelet infusion from transgenic donors [215]. This approach was successfully translated using an LV (2bF8LV) to transduce hematopoietic stem cells (HSCs), resulting in sustained platelet FVIII expression, survival after injury, and no anti-FVIII antibody formation.

Two ex vivo therapies based on LV are currently undergoing clinical trials. The therapy “Auto CD34 + PBSC transduced with lentiviral vector” (NCT05265767) uses FVIII-ET3 upregulated by the macrophage/monocyte-specific CD68 promoter. It involves isolating HSCs from the patient’s blood, transducing these cells with an LV vector, and further reinfusing the modified cells into the BM to achieve permanent expression of FVIII into the bloodstream by macrophages and monocytes (Figure 1). Some concern over immune response arises from the FVIII-ET3 variant’s porcine epitopes, but bone marrow preconditioning promotes immune tolerance induction (ITI) to the neoantigen. Results so far are encouraging: using an improved transduction protocol, 3 patients maintained stable FVIII activity above 15%, correlating with vector integration frequency [216].

An alternative strategy to ex vivo therapy with autologous HSC transplantation involves FVIII expression under the control of the platelet-specific ITGA2B gene promoter, as used in another clinical trial (NCT03818763) [217]. Platelet-targeted FVIII is protected from circulating inhibitors by isolation within platelet α-granules alongside endogenous VWF, rather than by intrinsic resistance to neutralization. Upon platelet activation at sites of vascular injury, FVIII-VWF complexes are released locally, supporting coagulation while limiting exposure to circulating inhibitors, as shown in a patient with a history of inhibitor formation [218]. This suggests that platelet-targeted ex vivo gene therapy may be successfully applied to patients with inhibitors, as demonstrated in a single patient with pre-existing nAbs [217]. The use of an alloantigen is considered a safer strategy, as the transgene does not contain xenogeneic epitopes. In this trial, the presence of nAbs is an inclusion rather than exclusion criterion, potentially widening the pool of patients who could benefit, though long-term follow-up will still be needed, given the insertional-mutagenesis risk that comes with LV.

### 4.3. Dual-Vectors AAV-Mediated Gene Therapy

AAV vectors have emerged as a promising approach to gene therapy due to their low immunogenicity and high tissue tropism [202]. One of the main obstacles to using AAV for HA is the great length of the factor VIII coding sequence, close to 7 kb for wild-type FVIII. Removing the B-domain, which is not required for FVIII’s functional activity, shortens the coding sequence (CDS) to 4.37 kb. Even so, that is only slightly under the AAV vector’s 4.7 kb capacity, leaving little space for the vector’s other functional elements [219].

Splitting the hFVIII coding sequence across two vectors is inspired by intracellular proteolysis, which naturally cleaves the FVIII protein into two polypeptides, the hFVIII heavy and light chains, as described above [220]. Burton et al. carried out the first study of separate AAV-mediated delivery of the full-length hFVIII heavy and light chains in 1999 [221]. Administering 3 × 10^11^ of each vector provided a light-chain concentration of 2–10 µg/mL, but activity was equal to only 230–430 ng/mL. The authors attributed this to the need for both FVIII chains to be secreted from within the same cell, meaning co-transduction by both vectors, and suggested, based on the imbalance observed, that the two chains are expressed with different efficiencies.

The chain imbalance was confirmed by later studies that described both human and mouse FVIII as being secreted 10- to 100-fold better than the heavy chain in vivo, despite equal RNA levels. In cFVIII, notably, this imbalance is less pronounced, with the light chain expressed only 4- to 8-fold, or 10- to 25-fold, higher [55,136,203,222,223,224]. FVIII activity in the case of the dual-chain (DC) delivery is therefore limited directly by the heavy chain concentration and by the need for both chains to be expressed within the same cell, since they can only combine into the heterodimer intracellularly [55,221,222]. Although the chains can re-associate in vitro after secretion, heavy-chain hFVIII secretion without co-expressed light chain is markedly less efficient than secretion of the full-length protein [54,225]. Various strategies were developed to overcome this imbalance and boost heavy-chain secretion: (1) replacing the FVIII chains with orthologous ones (canine FVIII, rat FVIII) [136,226,227]; (2) modifying the heavy chain separately; (3) screening the optimal ratio of heavy- to light-chain vectors [225,227]. One attempt to modify the hFVIII heavy chain (hFVIII-HC), the variant HC_HL_, added the a3 linker domain and raised hFVIII-HC expression 5-fold. Although the hFVIII-HC antigen level reached a healthy 100–200 ng/mL, activity amounted to only 0.1 IU/dL. Notably, activity rose roughly 3-fold when the vector ratio was 4:1 (HC:LC, with HC held constant) and the light chain was in 5-fold excess, compared with a more balanced chain ratio (9:1 HC:LC vector ratio). A result could be explained by inefficient in vivo chain re-association and by more effective heterodimer formation during secretion when the light chain is in abundance [225]. This raises further doubt on whether balanced secretion of the FVIII chains needs to be achieved for maximum FVIII activity.

Chain imbalance in vivo was successfully resolved by delivering cFVIII-HC and hFVIII-LC at a 4:1 ratio, giving 18- to 30-fold greater activity than delivering the two hFVIII chains. As the authors suggested, this mainly reflects cFVIII-HC’s high secretion efficiency without the necessity of the light chain expression since cFVIII-HC is secreted 20- to 30-fold better than the modified hFVIII-HC (HC_HL_) [227]. Consistent with earlier studies, the human FVIII heavy chain provided lower activity than its antigen level would predict, whereas cFVIII-HC activity roughly matched its antigen level. cFVIII has 3-fold higher specific activity, indicating the same underlying problem in incomplete re-association between heavy and light chains [140,224]. When observed, higher specific activity comes solely from properties of the cFVIII light chain, through the greater stability of the resulting heterodimer; however, it remains unclear why specific activity differs between the human and canine heavy chains. A more efficient re-association of the canine heavy chain with the light chain could be a feasible explanation. The chain imbalance may therefore be solved by improving heavy-chain secretion without the necessity of light-chain co-expression within the same cell, such as with cFVIII DC delivery [227].

Equimolar secretion of the FVIII heavy and light chains is required to provide plasma activity. In addition to a fold increase in the amount of the vector containing HC, another strategy was employed to enhance FVIII-HC expression through other vector regulatory elements, such as by using a stronger promoter [222]. However, in practice, heavy-chain expression is limited by secretion efficiency, and unsecreted protein that accumulates proves toxic to the cell, since heavy-chain expression alone can trigger ER stress and inhibitor formation [54,222,224,227,228,229]. This has increased importance for delivering the human FVIII heavy chain separately, given how poorly it is secreted on its own.

Several studies found that DC delivery provides FVIII activity at least comparable to, and sometimes greater than, SC delivery. This reflects the choice of more potent vector regulatory elements, made possible by the absence of a strict capacity limit, together with the cFVIII heavy chain’s more efficient secretion as a standalone polypeptide [220,223,224,230]. More recent reports, however, found the SC approach to be more efficient for cFVIII delivery [229,231]. The main limitation of cFVIII-based studies is precisely this greater secretion efficiency, which prevents extrapolation of the results obtained for both DC and SC approaches to orthologs, including hFVIII and mFVIII (murine FVIII) [140,229,231]. More efficient secretion in cFVIII also implies lower cellular stress for both delivery strategies.

Given these cFVIII-specific features, the results obtained so far offer little encouragement for implementation of the DC human FVIII delivery in clinical trials. Further development of AAV-mediated gene therapy for HA, therefore, had to return to a single-vector approach, with better optimization of both the transgene and the other vector’s elements.

### 4.4. AAV-Mediated Hemophilia A Gene Therapy

Once the FVIII-BDD coding region was packaged into AAV, researchers faced a further obstacle to gene therapy aimed at endogenous expression of the delivered FVIII copy. FVIII expression efficiency was 2–3 orders of magnitude lower than that of proteins of similar molecular mass [232,233]. This observation prompted efforts not only to shorten the coding sequence but also to boost FVIII expression and activity. Although the B-domain has no procoagulant function, it may promote FVIII secretion since B-domain deletion enhances mRNA levels 17-fold, but expression level boost is observed only by 30% [132]. Most current HA gene therapies therefore use codon-optimized FVIII-SQ variants, in which the B-domain is removed, but a 14-a.a. linker between the light and heavy chains is preserved (Table 1). Codon optimization is widely used in current HA gene therapies by employing various algorithms and resulting in CpG content [132,234,235]. Decreasing CpG content can reduce vector immunogenicity and improve the safety of therapy, though this is not always the case, since DTX201 contains 183 motifs [236,237]. Unmethylated CpG motifs are uncommon in mammalian DNA and may induce an innate host immune response [238].

Immune-related toxicity, particularly hepatotoxicity, is a well-known adverse event in AAV-based gene therapy. The process involves an initial innate immune response, followed by adaptive immune activation with antibody production and cytotoxic T-cell responses, which can damage transduced cells [239]. Clinically, this often manifests as elevated liver enzymes (aminotransaminases) and a subsequent decline in the therapeutic factor level, threatening both initial transduction success and long-term transgene expression durability [240,241]. Management strategies aim to control these immune responses. Immunomodulation (e.g., with corticosteroids) can be used reactively or prophylactically to suppress the immune response and preserve expression, though its efficacy varies [240,242].

**Table 1:** Hemophilia A AAV-FVIII gene-therapy programs in clinical trials.

Name	Developer/Sponsor	Actual Clinical Trial	Trans Gene	AAV Serotype	Dose (vg/kg)	Codon Optimization	Promoter	CpG Cassette (FVIII) Content	Platform	Results to Date	Ref.
BMN-270 (valoctogene roxaparvovec) FDA approved in USA (2023)	BioMarin	NCT03370913	FVIII-SQ	AAV5	4 × 10^13^ 6 × 10^13^ (phase 3)	+	HLP	10 (3)	Sf9	For 4 years (phase 3) Median activity: 13.5% (OSA); Mean ABR: mean 0.8 bleeds/year −82% reduction; 24/134 returned to prophylaxis; 90.3% ALT elevations; No treatment-related serious AE	[243]
SB-525 (giroctocogene fitelparvovec)-PF-07055480	Pfizer/ Sangamo	NCT04370054	FVIII-SQ	AAV6	9 × 10^11^ 2 × 10^12^ 1 × 10^13^ 3 × 10^13^ (phase 3)	+	CRMSBS2-TTRm	24 (9)	Sf9	For 15 months (phase 3): Mean activity: 135% (OSA); Mean ABR: 74% reduction (compared to prophylaxis); 74/75 experienced AEs with 7 serious AEs (to 104 weeks)	[244,245,246,247]
SPK-8011 (Dirloctogene samoparvovec)	Spark	NCT03003533	FVIII-SQ	SPK200 derived from LK03 (AAV3-derived)	5 × 10^11^ 1 × 10^12^ 1.5 × 10^12^ 2 × 10^12^	+	Modified TTR (TTRm)	14 (0)	HEK293	For 1 year (52 weeks): Median activity for 5 × 10^11^–5.4%, 1 × 10^12^–7.8%, 1.5 × 10^12^–4.1%, 2 × 10^12^–8.4%; Mean ABR: 91% reduction for 21 participants with sustained FVIII activity; 2 patients completely lost FVIII activity; one serious AE	[242,248,249,250]
SPK-8016	Spark	NCT03734588	FVIII-SQ ^1^	AAV-Spark	5 × 10^11^	NS	NS	NS	HEK293 ^1^	No representative data, see text	[251,252]
GO-8	UCL/ St. Jude	NCT03001830	FVIII-V3	AAV8	6 × 10^11^ 2 × 10^12^ 4 × 10^12^ 6 × 10^12^	+	HLP	14 (3)	HEK293T	For 3 years (2 × 10^12^ dose): Mean activity: ~15% (CSA); Mean ABR: NS	[132,251,253,254]
BAY-2599023/DTX201	Bayer/ Ultragenyx	NCT03588299	FVIII-SQ	AAVhu37	5 × 10^12^ 1 × 10^13^ 2 × 10^13^ 4 × 10^13^	+	E03.TTR	183 (173)	HeLa	No representative data, see text	[236,237]
BAX-888 (TAK 754)	Takeda	NCT03370172	FVIII-SQ	AAV8	2 × 10^12^ 6 × 10^12^ 1.2 × 10^13^	+	mTTR enhancer-promoter	36 (17)	HEK293	For 1 year: Peak activity: 3.8% and 11% (2 × 10^12^ dose); 54.7% and 69.4% (6 × 10^12^ dose) then loss to 12 months; All 4 participants resumed prophylaxis; All 4 participants faced treatment-related AE; 1 serious AE.	[255,256,257,258]
ASC618	ASC Therapeutics	NCT04676048	FVIII-ET3	AAV8	NS	+ Liver codon optimization	HCB	8 (0)	HEK293	No representative data, see text	[233,255,259,260]
ANB-010	BIOCAD	NCT06185335	SP-FIX- FVIII-SQ	AAV6	NS	+	NS	NS	NS	NS	[157], ^2^
BBM-H803 (BBM-002)	Belief BioMed	NCT06111638	FVIII-SQ	AAV XL32.1	1 × 10^13^	+ Human codon usage	LXP2.1	19 (11)	HEK293	NS	[261]
GS1191-0445 (continuation of GS001)	Gritelement Therapeutics Co., Ltd.	NCT06833983	FVIII-V3	AAV8	3 × 10^12^	+	Synthetic liver-specific (derived from canine *SERPINA1* and zebrafish *Albumin* genes)	10 (3)	HEK293	Median-dose cohort on 52 weeks: Activity: 77% (range, 5.9–324.2 IU/dL); ABR: 99.1% reduction	[262,263]
GS001	Institute of Hematology and Blood Diseases Hospital, China	NCT04728841			2 × 10^12^ 4 × 10^12^					Low-dose cohort (2 × 10^12^): Median activity: 7.2% on 52 week and 5.6% on 144 week; Median ABR: from 11 to 0.8 (92.7% reduction). High-dose cohort (4 × 10^12^): Median activity: 59.6% on 52 week and 42.6% on 104 week; Median ABR: 98.4% reduction; All patients are free from prophylaxis.	[264,265]
ZS802	Institute of Hematology and Blood Diseases Hospital, China	NCT05523128	modified FVIII-SQ (FVIII-h/megabat)	AAV5	2 × 10^13^ 6 × 10^13^	NS	Em-hAATsh	NS	NS	NS	[159,266]
NGGT003	Suzhou Nggt Biotechnology Co ltd	NCT06238908	FVIII-L1	AAV8	4 × 10^11^ 1 × 10^12^ 2.5 × 10^12^	NS	Modified TTR (TTRm)	NS	NS	NS	[154]

^1^ Data were taken from the extrinsic publications. ^2^ Personal communication. +—is codon-optimized; NS—Not Specified.

### 4.5. The Liver as a Target for Hemophilia A AAV-Mediated Gene Therapy

Although FVIII is naturally expressed by liver sinusoidal endothelial cells (LSECs), its content in the liver is less than 3%, making these cells a difficult target for vector delivery and transgene expression [267]. Therefore, AAV-mediated gene therapies are generally directed at the liver (including HA gene therapies), specifically targeting the most abundant cell type, hepatocytes, which are responsible for the synthesis of the majority of the liver’s secreted proteins [268]. The interaction between different liver cells enables the development of immune tolerance to the expressed protein [201,269].

The liver’s inherent tolerogenic nature was first recognized in 1969 with successful MHC-mismatched organ transplants [270]. This capacity is now a cornerstone of hepatic gene therapy and is explained by different mechanisms. Firstly, the liver’s diverse population of antigen-presenting cells (APCs) is critical to induce tolerance. Dendritic cells (DCs), such as liver-resident macrophages and Kupffer cells (KCs), express low levels of MHC II and co-stimulatory molecules (CD40, CD80/86) and secrete anti-inflammatory mediators like PGE2 and IL-10, which promote immune tolerance due to induction of Treg and incomplete T cell activation [271,272,273,274,275,276]. Also, DCs and KCs induce anergic T cells or delete effector T cells through the PD-L1/PD-1 pathway and via indoleamine 2,3-dioxygenase (IDO) secretion [273,277,278,279,280].

Non-conventional APCs (liver sinusoidal endothelial cells (LSECs), hepatic stellate cells, and hepatocytes) are also efficient at antigen presentation and support tolerogenic effects [281,282]. LSECs are among the first cells to contact blood-borne lymphocytes. They can convert conventional T cells into FoxP3+ Tregs in a TGF-β-dependent process and are also responsible for generating type 1 regulatory (Tr1) cells that produce IL-10 and IL-4 [283,284]. This ability to induce different types of regulatory cells makes LSECs a pivotal player in hepatic tolerance. Moreover, LSECs are the cells that naturally produce FVIII in the body [285], unlike FIX, which is produced in hepatocytes [24,286]. In conjunction with a high potency of tolerance induction, these cells are attractive targets for AAV delivery in the context of gene therapy.

Lastly, hepatocytes, the most abundant liver cell type (~80%), also possess antigen-presenting capabilities [287,288,289]. Under steady-state conditions, they express low MHC-I and no detectable MHC-II, though MHC-II can be induced during inflammation [290,291,292]. Circulating T cells can interact directly with hepatocytes through the fenestrations in the LSEC layer (https://doi.org/10.1002/hep.21378). The presentation of antigens by hepatocytes can induce FoxP3+ Tregs through both Notch-dependent and TGF-β-dependent conversion pathways [293]. Furthermore, transduced hepatocytes upregulate FasL, which engages Fas on activated lymphocytes, leading to their direct deletion and contributing to the clearance of antigen-reactive cells.

In summary, due to the features of antigen presentation processes in the liver, AAV delivered this way induces regulatory T cells, as well as antigen-specific CD4+ and CD8+ Tregs [294,295,296]. Although the expansion of the latter was shown only in mice in allogeneic MHC I (H-2Kd) and MOG (myelin oligodendrocyte glycoprotein) AAV-based gene transfer [297,298]. Practically, data obtained by preclinical and clinical studies support the tolerogenic effect of the transgene in AAV-based gene therapy [295]. As for hemophilic patients, in all AAV-based gene therapy clinical trials, the level of FVIII inhibitors did not increase after treatment [240]. One patient showed detectable FVIII-specific TAbs (total antibodies, not nAbs) after Valoctocogene Roxaparvovec treatment, but they did not have a deleterious effect on the safety or efficacy of therapy [299].

### 4.6. AAV-Mediated Gene Therapies Results

#### 4.6.1. Valoctocogene Roxaparvovec-Rvox (BMN270)

Roctavian (valoctocogene roxaparvovec-rvox) became the first, and so far only, approved gene therapy for HA (currently withdrawn). Its AAV5 vector carries a codon-optimized hFVIII-SQ sequence under the HLP promoter and was originally developed by St. Jude before being licensed to BioMarin [132,251]. The pilot dose-escalation trial, BMN270-201 (NCT02576795), found that low and medium doses (6 × 10^12^, 2 × 10^13^, 4 × 10^13^ vg/kg) provided disproportionately low activity of <3%, while the high dose (6 × 10^13^ vg/kg) caused a high mean activity of 64% (CSA) over the first year. Activity then declined rapidly through years 2 and 3 (mean 36% and 33%). The activity decline slowed in later years, reaching 12.6% (median 5.1%) by year 7, which accounts for the return of clinical symptoms seen in some patients [251,300,301,302]. A phase 3 trial demonstrated a similar, rapid decline in activity by the second year, with 40% of participants falling below 5 IU/dL FVIII activity by the fourth year [243,303]. In the 6 × 10^13^ vg/kg group, mean ABR fell to 0.75 with a 96% reduction from baseline, although 2 patients returned to prophylactic treatment owing to severe or frequent spontaneous bleeding. Mean FVIII activity was 12.6% (median 5.1%) in the 6 × 10^13^ vg/kg group at year 7 and 5.2% (median 4.5%) in the 4 × 10^13^ vg/kg group at year 6, both before patients resumed prophylaxis. By year 7, some patients in both groups had a treatment-related AE: one patient in the 6 × 10^13^ vg/kg group experienced hepatomegaly lasting 35 weeks, while another patient with a history of fatty liver disease in the 4 × 10^13^ vg/kg group experienced grade 1 splenomegaly alongside worsening grade 1 hepatic steatosis. Both cases were resolved following their last examination, but these incidents still raise questions about the therapy’s long-term safety [302].

In the phase 1/2 trial, patients in the 6 × 10^13^ vg/kg dose cohort showed no clear link between declining FVIII activity and elevated ALT, since the transaminase elevations remained asymptomatic through the first year [304]. However, in Phase 3 clinical trials, the decline in activity was associated with alanine aminotransferase (ALT) elevations caused by transaminitis and immune-mediated hepatitis [305,306]. The long-term treatment with glucocorticoids successfully resolved transaminitis and halted the activity decline [307]. Although expression was stabilized after transaminitis, long-term preservation of activity is not ensured.

#### 4.6.2. GO-8

The UCL-funded GO-8 trial (NCT03001830) uses a vector similar to BMN-270. GO-8 differs slightly in replacing the SQ linker with the V3 variant, which carries additional N-glycosylation sites. In preclinical mouse studies, FVIII-V3 provided 3-fold higher expression than FVIII-SQ. Vector capacity limitations during early preclinical work also led to a new liver-specific promoter, named HLP (hybrid liver promoter), which was shorter than LP1 while offering similar expression levels [132,251,308]. Unlike BMN-270, the GO-8 vector is manufactured on the HEK293T platform and was tested clinically at four doses: 6 × 10^11^, 2 × 10^12^, 4 × 10^12^, and 6 × 10^12^ vg/kg. In the high viral load (≥4 × 10^12^ vg/kg) groups, elevated ALT appeared in 10 of 12 patients, with complete loss of transgenic protein in one participant. Interestingly, the intermediate 2 × 10^12^ vg/kg group maintained consistently higher activity than the 4 × 10^12^ vg/kg group over 5 years [253]. The researchers report greater stability of FVIII activity in GO-8 than in the BioMarin trials, though it is worth noting that activity in the 2 × 10^12^ vg/kg group peaked lower than for BMN-270, while mean FVIII activity over 3 years was still higher with BioMarin’s therapy. The data for GO-8’s highest dose group, 6 × 10^12^ vg/kg, have not yet been reported [251,254]. Even so, this therapy achieved an order-of-magnitude reduction in viral load while maintaining therapeutic activity. This improvement may reflect greater FVIII-V3 expression and/or better transduction from the more liver-tropic AAV8 serotype, produced on the HEK293 rather than the sf9 platform [251].

#### 4.6.3. SPK-8011

The Spark Therapeutics product, SPK-8011, aimed to reduce cellular immune response to the AAV capsid by lowering viral load [242]. To boost vector efficacy, SPK-8011 used a new synthetic AAV serotype, SPK200, derived from LK03; the codon-optimized FVIII-SQ transgene is upregulated with the minimal mouse TTR promoter, with the HNF3 transcription-factor binding site modified to a higher-affinity variant [309]. This modification is reported to enhance in vivo FVIII expression 4-fold [310]. Using a minimal promoter without an enhancer also created space for a synthetic intron from the human EF-1a gene upstream of the CDS, which could potentially boost FVIII expression.

In the pilot SPK-8011-101 study (NCT03003533), all 25 patients had treatment-related AEs within a year, including one serious AE requiring hospitalization due to 3- to 4.9-fold ALT elevation. The 1.5–2 × 10^12^ vg/kg groups showed a cellular immune response to the SPK200 vector that was resolved with glucocorticoids, with 4 patients having serious AEs from these immunosuppressants. At the lower doses (5 × 10^11^ and 1 × 10^12^ vg/kg), FVIII activity showed no rapid decline after reaching its peak within 12 weeks and stayed stable for at least 3 years, in contrast to the higher-dose groups (1.5 × 10^12^ and 2 × 10^12^), where activity peaked at 55.1% and 38.7% (median OSA) before falling rapidly to 4.1% and 8.4% by week 52. It is worth noting that 2 patients in the 2 × 10^12^ vg/kg group lost FVIII activity entirely within the first year owing to a cellular immune response to the vector and were excluded from the analysis along with a third patient who had not yet reached the 52-week cut-off mark [248].

Spark paused its phase 3 trial (NCT06297486) despite modest, yet stable, FVIII activity in the mild HA range, averaging 7.4% over 4 years [248]. A phase 2b trial is planned instead, using the bioengineered, AAV3-derived (LK03 subtype) capsid together with the FVIII-QQ variant characterized by improved hemostatic potency that may contribute to better hemostasis with modest FVIII levels [16]. Patient recruitment for the SPK-8011QQ trial is ongoing (NCT07226206).

#### 4.6.4. SPK-8016

The SPK-8016-101 trial (NCT03734588) was designed to assess the treatment of patients with nAbs to FVIII. To evaluate the safety and efficacy of the therapy, a phase 1 trial was conducted in patients without active inhibitors or a history of inhibitor formation, using a single vector dose. Only 4 patients received 5 × 10^11^ vg/kg of a vector with its serotype, promoter, and transgene remaining unpublished. The trial ended early, even though dose elevation up to 1 × 10^12^ vg/kg was planned for patients with inhibitors, as with SPK-8011. For this study, preliminary results indicated an increase in activity of 6.2–21.8% into mild HA by week 52. However, the activity was unstable in 3 patients who received oral corticosteroids as a means of overcoming the immune response [311]. Seven bleeding episodes occurred in the first year, with six traumatic and one spontaneous. The study is currently suspended due to the lower-than-expected impact [312].

#### 4.6.5. PF-07055480 (SB-525)

Like most HA gene therapies, giroctocogene fitelparvovec utilizes FVIII-SQ under a liver-specific promoter. PF-07055480 differs from other therapies, as it utilizes an AAV6-based vector (produced on the sf9 platform) with a new liver-specific promoter, CRMSBS2-TTRm. The pilot dose-escalation trial (NCT03061201) aimed to find the vector dose that would achieve FVIII activity within the 40–100% range.

About two-thirds of preliminary patients failed to meet the inclusion criteria, leaving just 11 enrolled, across 4 dose groups: 2 patients at 9 × 10^11^ vg/kg, 2 at 2 × 10^12^ vg/kg, 2 at 1 × 10^13^ vg/kg, and 5 at 3 × 10^13^ vg/kg. Anti-AAV6 antibodies accounted for most exclusions (15 persons). Only 5 patients reached activity above 5% by the end of year 1, 1 in the 1 × 10^13^ vg/kg group and 4 in the 3 × 10^13^ vg/kg group. In the highest-dose group, activity fell 40% by year 2, to 38.9% (mean OSA), though one patient with activity near 100% raised the mean considerably. Of two patients with supraphysiological peak activity (169% and 187%), one experienced a rapid decline in activity between weeks 64 and 128. However, despite these high peaks, no thrombosis or inhibitor formation was seen in either patient. By year 5, activity in the highest-dose group showed a mean OSA of 41%. All patients had treatment-related AEs, with serious AEs in 2 patients across the two highest-dose groups [244].

The phase 3 AFFINE trial (NCT04370054) demonstrated a similar pattern: around half the patients (at 3 × 10^13^ vg/kg) reached peak CSA above 150%, requiring anticoagulant therapy until FVIII activity returned to physiological levels. Preliminary results in the first 50 participants show a 74% reduction in mean ABR relative to prophylaxis from week 12 to month 15 post-infusion with FVIII activity at 135% (mean OSA); current data show activity holding at 72.9% (mean OSA) by year 2 in 29 patients [16]. Even so, Pfizer chose not to pursue a biologics license for the therapy, and rights have reverted to Sangamo [313].

In the AFFINE clinical trial, a case of nAbs formation against FVIII was reported for the first time in a single patient following AAV gene therapy. Total anti-FVIII antibodies were detected on day 219, while nAbs were reported from day 333. In parallel, FVIII activity declined rapidly by day 368, and from day 487, the activity and antigen levels dropped below the limit of detection. The authors suggest that the most likely explanation for the loss of expression is recurrent transaminitis, since FVIII activity was partially restored with the treatment of corticosteroids. Liver biopsy from this patient could help reveal the underlying reasons for the transaminitis. However, the specific mechanism of nAb formation also remains unclear [314].

#### 4.6.6. TAK-754 (BAX 888)

TAK-754 was developed using a CpG-depleted, codon-optimized FVIII-SQ under the mouse TTR enhancer/promoter within an AAV8 vector. Codon-optimization enhanced in vivo FVIII expression 74-fold over wild type while also reducing CpG content [255]. The clinical trial involved 4 patients across two dose groups, 2 × 10^12^ and 6 × 10^12^ vg/kg. Regardless of dose, transaminitis-driven loss of FVIII activity emerged over 12 months, leading to the trial being halted. This loss occurred without impaired liver function and could not be prevented with glucocorticoids, and its cause remains unknown. Since peak FVIII activity never exceeded physiological levels, a subsequent fall was unexpected. In the HB trial BAX-335, a similarly rapid drop in FIX expression was linked to a cellular immune response driven by high CpG content. In TAK-754, CpG content had already been intentionally minimized, and since FVIII expression loss was not rapid, CpG content is likely not the underlying reason.

#### 4.6.7. BAY 2599023 (DTX201)

DTX201 uses the AAVhu37 serotype from the hepatotropic clade E family, chosen for demonstrating the lowest immunogenicity in mouse and non-human primate (NHP) studies [237]. Its vector is distinguished by the E03.TTR promoter, a combination of the core human TTR promoter with the mouse TTR enhancer [315]. Despite the long-running clinical trial NCT03588299, published results remain sparse, with no data yet available for the highest dose group (4 × 10^13^ vg/kg), which was added later. The therapy is reported to sustain expression for at least 23 months across three doses, despite its high CpG content, and all patients with the 1 × 10^13^ and 2 × 10^13^ vg/kg doses discontinued prophylaxis within 6–12 weeks post-vector administration. Treatment-related AEs occurred in 5 of 9 patients, with one serious AE and elevated ALT in the 2 × 10^13^ vg/kg group [316].

#### 4.6.8. ASC618

Preclinical development of ASC618 focused on shortening the expression cassette while raising expression efficiency. This led to a new synthetic liver-specific promoter, HCB (hepatic combinatorial bundle), which, despite being only 146 bp in length, provided 14-fold higher in vivo FVIII expression than HLP. A second approach used a chimeric human/porcine FVIII variant, FVIII-ET3; codon-optimized for highly expressed liver genes (LCO) raised in vivo expression a further 7-fold. The obtained expression cassette, at 4912 bp, is the smallest among current AAV vectors for HA [233]. The clinical trial is currently ongoing (NCT04676048); the doses being used have not yet been disclosed, though the vector’s apparent potency should translate into a lower viral load. In preclinical NHP studies, AAV8-HCB-ET3-LCO provided 0.3 IU/mL expression at a minimal dose of 5 × 10^11^, though inhibitors appeared from week 6 [259]. A loss of activity in a single animal that received a higher dose of vector (1.5 × 10^12^ vg/kg) without the formation of inhibitors may raise concerns about the possible occurrence of a similar response in patients in clinical trials. Moreover, it was recently reported that the ASC618 clinical trial was suspended due to the high titers of nAbs developed in two patients. The most likely cause appears to be the presence of xenogeneic epitopes on the FVIII-ET3 protein’s surface [317].

#### 4.6.9. GS1191-0445 (GS001)

GS1191-0445 (formerly GS001) was the first HA gene therapy to enter clinical trials in China. Like GO-8, it uses the AAV8 serotype to deliver a functional copy of FVIII-V3 [132,265], though its other regulatory elements differ: the promoter combines a modified canine Alpha-1 antitrypsin sequence with the zebrafish *Albumin* gene promoter, and its polyadenylation signal is derived from the common vole polyomavirus KS13 strain. Together, these modifications make the GS001 expression cassette roughly 3-fold greater potency than HLP-codop-FVIII-V3 (GO-8), both in vitro in Huh7 cells and in vivo in a mouse model [265].

Only 12 of 40 preliminary patients were enrolled in the phase 1 trial (NCT06833983), with the main exclusion criteria being seropositivity (21 participants); 12 remaining participants were split into a low-dose cohort (2 × 10^12^ vg/kg, 6 patients) and a high-dose cohort (4 × 10^12^ vg/kg, 6 patients). In the low-dose cohort, activity peaked at 80.4% (median OSA) by weeks 5–6, then fell sharply to just 7.2% (median OSA) by the end of year 1 and 5.6% by week 144. Patients who received tacrolimus alongside prophylactic prednisolone maintained higher activity over the 144 weeks than those without immunosuppression.

In the high-dose cohort, activity peaked disproportionately higher at 288.2% (median OSA) by weeks 6–7, meaning all 6 patients in this cohort experienced an activity level above 150% as an adverse event. Despite this higher peak, the percentage decline afterwards was substantially smaller than in the low-dose cohort: median activity stood at 59.6% by the end of year 1 and 42.6% by year 2 (week 104), with one patient maintaining a fully healthy 98.8% activity. The extent of decline during the year correlated strongly with the number of AAV-specific T cells present on day 7 post-administration. Upon the addition of tacrolimus to prednisolone, there was a reduction in CD8+ cells without any substantial effect on the CD4+ cells, limiting this AAV-specific T-cell response [265]. Prophylactic tacrolimus, however, brings its own side effects. A phase 3 trial (NCT06833983) is currently ongoing with a medium dose of 3 × 10^12^ vg/kg, apparently seeking a middle ground between adequate FVIII activity and a lower risk of adverse reactions; this group shows activity of 77.0% (range 5.9–324.2 IU/dL) by the end of year 1, with a 99.1% reduction in ABR [318].

#### 4.6.10. ANB-010

Biocad’s ANB-010 vector is in an early clinical dose-escalation trial that has already enrolled 50 participants. It is distinguished by the use of the factor IX signal peptide, which raises in vitro FVIII expression 4.3-fold in cell culture (US20260242826; personal communication) [157]. ANB-010 relies on the AAV6 serotype, as SB-525 does, reflecting this trial’s patient inclusion criteria (NCT06185335).

#### 4.6.11. ZS802

ZS802 therapy delivers a modified FVIII-SQ variant via AAV5, under a new and exceptionally short synthetic liver-specific promoter, Em-hAATsh, which gave 10-fold higher luciferase activity in the Huh7 line than the previously used HLP (WO2024230802A1) [318,319]. The FVIII-SQ transgene (Megabat) itself was modified with a.a. substitutions designed to raise its secretion efficiency 3-fold (US20240254197A1) [159]. Despite the cassette’s expected high efficiency, the clinical trial plans fairly high vector doses, 2 × 10^13^ and 6 × 10^13^ vg/kg, similarly to valoctocogene roxaparvovec (NCT05523128), which may raise the risk of supraphysiological FVIII activity.

#### 4.6.12. BBM-H803 (BBM-002)

BBM-H803 is still at an early clinical stage with no published results. The experimental plan was to inject a single dose of 1 × 10^13^ vg/kg of a vector designed with the new synthetic serotype AAVXL32.1 (AAV843), previously used in the BBM-H901 therapy for HB, currently approved in China (US20260035715A1) [320,321]. Its codon-optimized, CpG-depleted FVIII-BDD transgene is upregulated with a new, short synthetic liver-specific promoter, LXP2.1 (188 bp), constructed from a combination of hepatocyte transcription-factor binding sites [261,309].

## 5. AAV Gene Therapy Discussion

In clinical practice, stable factor IX activity of more than 30% has been achieved in patients with HB, which remains challenging for patients with HA. The rapid loss of FVIII activity over time remains the major obstacle to safe, effective gene therapy for HA. A recent meta-analysis of clinical trial data found that FVIII activity falls, on average, to 55.8% of its peak by year 2 post-administration, compared with 95.7% for HB [322]. Exact mechanisms for activity loss remain unknown, and the various hypotheses proposed do not always agree between studies, most likely reflecting differences in study design. The mechanisms most often suggested include:

1. A capsid-specific T-cell response that eliminates transduced hepatocytes depending on the AAV serotype used, the dose, and the empty-to-full capsid ratio [240,323,324,325,326,327];
2. Transgene silencing and epigenetic modification of vector DNA, potentially influenced by the AAV production platform and the vector’s oversized status [328];
3. ER stress and UPR, driven by low FVIII secretion efficiency [168,237].

In addition, the efficacy of therapy is further affected by a variety of factors not directly related to the vector. These factors are referred to as host-mediated post-transduction mechanisms of transcription, translation, and secretion, which are influenced by the patient’s liver health, metabolic status, tolerance to immunosuppression, chaperone expression, and others [285,329,330].

Yet the first two mechanisms ought to be relevant to other gene therapies, including for HB, where stable, long-term FIX activity is instead observed while using the same serotype and manufacturing platform, but not the oversized status [331]. In fact, AMT-060, an AAV5-mediated gene therapy for hemophilia B and the ancestor of Hemgenix, manufactured with the sf9 platform, provides stable expression for at least 8 years [332].

No AAV assembly platform has emerged as clearly superior for in vivo administration in humans, but it is worth noting that therapies currently in clinical trials tend to use higher doses when the vectors are produced on the sf9 platform. This may point to lower transduction efficiency relative to other platforms, consistent with findings in humanized mice carrying a human liver [333]. This lower infectivity may relate to the lower VP1 content found in sf9-produced capsids, which use the baculovirus system [251].

Overall, oversized vectors provide lower transgene expression than standard-sized ones at equivalent transduction efficiency, while producing oversized AAV vectors on the sf9 platform generates more fragmented genomes than on HEK293 [328]. Preclinical studies show a dependence of long-term expression on the presence of full-length genomes, while genomes lacking ITRs are largely degraded [268]. The mechanisms behind expression loss, however, appear to depend on the specific combination of manufacturing platform and oversized status. For oversized vectors made on HEK293, transgene expression correlates with vector genome number, whereas for sf9 it correlates instead with RNA level and the degree of vector DNA methylation [230,328]. In fact, post-transduction vector methylation in liver cells is more common for the sf9 AAV manufactured with the sf9 platform, which is another factor to be considered for developing new therapies [328].

ALT level, the marker of hepatotoxicity, elevation occurred in half to 90% of patients (depending on the threshold criteria) through the first four years of follow-up after treatment with HA gene therapy, which raises safety concerns due to the association of acute transaminitis with short-term decline in circulating FVIII activity [251,304,334]. However, the specific mechanism linking elevated ALT levels to a loss of FVIII activity remains unclear. Nevertheless, corticosteroids are the first-line treatment for transaminitis, but their use requires a highly individualized approach based on the latest clinical recommendations. Furthermore, experts suggest that a patient who is not a good candidate for immunosuppression may not be a good candidate for gene therapy [330]. Reactive corticosteroid use is recommended for early ALT elevations, with the treatment finished as quickly as appropriate ALT levels return to <1.5× baseline or <ULN (upper limit of normal), and withdrawn if immunosuppression fails to prevent FVIII decline [305]. A successful outcome was demonstrated for six years of efficacy and safety of FVIII expression after valoctocogene roxaparvovec following personalized immunosuppression [307].

However, it remains unclear how markers of hepatotoxicity are actually related to the rapid loss of FVIII expression. ALT elevation occurs twice as often in AAV-mediated gene therapy for HA as for HB, pointing to greater hepatotoxicity of the therapy [305]. Only 17% of patients with HB treated with the AAV5-FIX vector experienced liver toxicity signified by liver enzyme elevations within 6 months, and only ~2% thereafter for the next 4.5 years from gene transfer. This observation suggests the presence of an additional factor contributing to liver toxicity in HA gene therapy, aside from cytotoxic T-cell responses against the AAV5 capsid. The plausible explanation of increased hepatotoxicity is the intracellular expression of FVIII and following ER stress [335].

However, until recently, no evidence of ER stress had been observed in liver biopsies from humans and mice treated with BMN-270 [301]. The absence of ER stress was attributed to moderate levels of transgene expression, as observed earlier in a mouse study [336]. However, in humans, the absence of signs of ER stress appears to be related to the fact that the biopsies were obtained years after gene transfer or that not every ER-stress marker was assessed [268]. Although two cases of mild immune-mediated hepatitis with signs of ER stress and ALT level>ULN have recently been reported 9 and 27 weeks after valoctocogene roxaparvovec treatment [306]. Both patients had significantly elevated expression of BiP, the spliced XBP1 isoform (XBP1s), and CHOP, which are modulated downstream of the ATF6, IRE1, and PERK pathways of UPR activation, respectively, which was consistent with the marked expansion of the ER observed in TEM analysis. A depletion of CD4+ T cells was observed in a patient (index case) with a high ALT level (633 U/L), as evidenced by the increased content of CD39PD-1-positive cells. CD8+ cell enrichment indicated the development of active cytotoxic activity during elevated ALT levels. Conversely, Tregs and circulating follicular T helper cells (cTFh), key regulators of the humoral immune response, had been reduced before ALT returned to normal levels.

This recent report highlights the high diagnostic value of biopsy analysis for assessing the safety and efficacy of HA gene therapies. Although immune suppression with corticosteroids appears to be an appropriate therapeutic approach in clinical practice, the high variability in HA gene therapy outcomes, which is independent of corticosteroid use, emphasizes the necessity of developing an evidence-based personalized approach for patient management [306].

Across all earlier, recent, and current clinical studies, results demonstrate an initially high peak of FVIII activity and suggest it as the main predictive indicator of rapid expression loss, even though the precise mechanism remains undetermined [10,158,211,337]. Unlike valoctocogene roxaparvovec (Roctavian) and giroctocogene fitelparvovec (SB-525), both of which reached near-normal activity before losing it sharply, therapies with a lower peak activity (in the mild-to-moderate hemophilia range) have sustained stable activity for 2–5 years post-injection [254]. This matches earlier preclinical findings in dogs, where a low initial activity level held stable at a median of 12.7% (OSA) for 8.2–12 years after administration [230].

High peak activity has driven nAbs formation in animal models; however, this was not observed to occur in humans until recently. Epidemiological data do show, however, that thrombosis risk rises more with high FVIII levels than with high FIX levels [240], and a case of thrombosis was recorded in a patient on the highest vector dose (3 × 10^13^ vg/kg) in the PF-07055480 trial (NCT04370054). This trial also reported the first case of nAbs formation against FVIII-SQ coupled with a complete loss of FVIII activity and antigen in a patient with an extremely high peak FVIII:C of 6.25 IU/mL. However, both the causes of the loss of FVIII expression and the origin of the antibodies remain unclear [314]. At the same time, higher transgene expression may promote immune tolerance, further complicating the choice of therapeutic activity window when designing a trial [269]. Efforts are therefore underway, so far in animal models, to predict this FVIII activity window more precisely, aiming for stable activity alongside a minimal risk of nAbs development [338].

Currently, capsid-specific antibodies, FVIII-specific nAbs, and liver disease all exclude patients from HA gene therapy. The first of these might eventually be addressed with new products built on non-cross-reactive AAV serotypes. Additionally, a better understanding of immune tolerance could open effective therapies to patients with inhibitors. However, hepatotoxicity in patients with liver diseases remains a more complex problem to address [237,339]. Lowering viral load improves safety and reduces hepatotoxicity but also demands more potent vectors to achieve the same therapeutic effect. However, raising FVIII expression in a smaller pool of transduced cells is not necessarily desirable either, as stronger promoters in HA drive intracellular FVIII accumulation, triggering UPR and nAbs formation [315,338,340]. This could also eliminate the already-small population of transduced hepatocytes, wiping out FVIII expression altogether. Even so, sustaining high vector doses is not a safe strategy for patients with liver disease. One way through this may be to reduce ER stress and UPR by developing new FVIII variants with more efficient secretion [113]. Examining such variants in clinical trials would also help settle whether low secretion efficiency and ER stress could explain the rapid loss of FVIII activity when compared relative to HB gene therapies.

Preclinical FVIII variants such as R1645H and its derivatives, along with FVIII-ET3, FVIII-X5, and the ancestrally reconstructed An-53, could improve transgene-derived FVIII production and, through more efficient secretion and potentially reduced UPR activation, the safety of therapy as well [142,150,338]. However, improved secretion alone does not ensure sustained expression. Despite providing 3-fold higher expression than FVIII-SQ, FVIII-V3 has not solved the problem of rapid activity loss or produced long-term stable expression in clinical trials [10,132,251]. If the cause of this activity loss, which is being debated, turns out not to be intracellular hepatocyte stress from FVIII expression but primarily a function of circulating FVIII levels, then variants with improved procoagulant properties could boost FVIII:C while keeping FVIII:Ag levels within the mild-to-moderate HA range [135,150,158,172].

The use of chimeric FVIII proteins retaining only the minimal set of substitutions required for enhanced expression has shown promise in combination with liver-directed gene delivery. However, the first clinical case of high-titer neutralizing antibody development has been reported following AAV-mediated liver-directed gene therapy with ASC618, which encodes FVIII-ET3 [317]. In preclinical studies, inhibitor development had previously been observed in mice receiving high-dose AAV-ET3 (ASC618) [341]. Inhibitors were also detected from week 6 onward in nonhuman primates receiving a low dose of AAV8-HCB-ET3-LCO (5 × 10^11^ vg/kg), which achieved FVIII activity levels of approximately 30% of normal [259]. Furthermore, in a separate NHP study using AAV vectors encoding a different engineered FVIII variant incorporating orthologous substitutions (FVIII-SQ Ver.4), 6 of 7 animals developed neutralizing antibodies [149]. The lack of immune tolerance may reflect the fact that the liver primarily supports peripheral tolerance mechanisms involving LSECs, Kupffer cells, and Tregs but does not itself mediate thymic negative selection [195,342]. Foreign epitopes introduced by orthologous substitutions may therefore be recognized by high-affinity T-cell clones that have escaped central deletion. Under conditions of high-dose vector administration, rapid expression, and cellular stress-mediated inflammation, peripheral suppression may be insufficient to prevent an immune response—pre-existing cross-reactive memory T cells can rapidly mount a secondary response [195,341,342,343]. In addition, foreign peptides are more efficiently presented by APCs, and the absence of tolerance mechanisms specific to self-antigens further enhances the immune response [195,342,343]. Nevertheless, foreign substitutions are not the only potential source of FVIII immunogenicity. In the PF-07055480 clinical trial using FVIII-SQ, one patient was reported to have developed neutralizing antibodies [314].

Antigen handling of FVIII itself may also contribute to its immunogenicity, as demonstrated in the spleen. Limited uptake by splenic red pulp macrophages may allow FVIII to bypass potentially tolerogenic antigen-handling pathways, whereas its efficient delivery to conventional dendritic cells promotes CD4^+^ T-cell activation and subsequent antibody responses. These processes were investigated in murine models [344].

Interestingly, in the HSC approach (NCT05265767), none of the five patients developed inhibitors despite the use of the same FVIII-ET3 transgene [216]. Taken together, these observations suggest that neither improved secretion nor sequence engineering alone is sufficient to ensure a durable therapeutic response. A successful approach may need to combine efficient secretion and improved procoagulant activity with a delivery and expression context that supports immune tolerance. In this regard, the potential importance of conditioning the immune environment before administration, as employed in HSC-based delivery, is of particular interest.

Myeloablative conditioning with treosulfan and fludarabine, used in ex vivo approaches, depletes mature T and B lymphocytes, including memory populations, thereby establishing conditions for immune reconstitution [216,345]. Transduced CD34^+^ HSCs reconstitute the immune system, and their progeny mediate thymic presentation of proteins bearing orthologous substitutions as a self-antigen, promoting negative selection of high-affinity T cells and induction of Tregs [216,343,345]. The CD68 promoter restricts transgene expression to myeloid cells, including monocytes and macrophages, which function as tolerogenic APCs, reinforcing peripheral tolerance while avoiding antigen presentation by the HSCs themselves [216,346,347]. In contrast, AAV trials rely on pharmacological immunosuppression with corticosteroids, MMF/tacrolimus, or sirolimus/rituximab to mitigate hepatotoxicity and T-cell responses. However, these regimens do not reliably prevent anti-AAV antibody formation or induce thymic tolerance [348,349]. More intensive lymphodepleting or antibody-directed interventions, including rituximab plus cyclosporine, ATG/MMF/tacrolimus, bortezomib plus anti-CD20, rapamycin nanoparticles, IgG-degrading enzymes, and plasmapheresis, can overcome pre-existing neutralizing antibodies and, in some cases, enable AAV readministration, although their effects are transient and associated with infectious risks [348,349,350]. For highly immunogenic FVIII-ET3 delivered by AAV vectors, the immunomodulatory interventions described above are likely to delay or attenuate immune responses rather than establish durable tolerance, which may require strategies that induce central tolerance or the use of a less immunogenic transgene combined with low-dose delivery or gradual expression [195,216,342,345,350,351].

The idea of AAV-mediated expression of emicizumab may seem attractive, but its implementation poses numerous design challenges: the CDS needs to be shortened, either through equimolar synthesis or efficient secretion, and correct assembly of the heavy and light chains into a functional heterodimeric antibody is required for the DC delivery strategy [336]. A further challenge is the lack of outcome measures, other than the actual bleeding rate, that can reliably predict hemostatic efficacy and the risk of thrombotic adverse events.

HA gene therapy promotes potential long-lasting effects and hopefully is required once per life. AAV-based therapy can lead to immune tolerance to the transgene that was shown in liver-targeted therapies [200,201,352,353]. This approach is supposed to overcome inhibitor formation problems in patients with HA. However, the rapid loss of FVIII activity in valoctocogene roxaparvovec raised early cost-effectiveness questions, mainly: when comparing relative health benefits to its closest competitors (e.g., emicizumab), how useful is the AAV-mediated gene therapy for HA? In their study, Renske et al. aimed to incorporate and quantify novel measures of value such as treatment durability, maximum value-based price (MVBP), and break-even time (i.e., time until benefits begin to offset upfront payment) for valoctocogene roxaparvovec. Valrox’s mean break-even time was 8.03 years compared to FVIII concentrates, or 5.68 years compared with emicizumab [322,354]. When comparing treatments for HB, Hemgenix, the mean break-even time was found to be 10.6 years when compared to recombinant FIX [355].

Almost all of the listed clinical trials exclude patients with active FVIII inhibitors or a history of FVIII inhibitor formation. Potential recurrence of inhibitors in patients with a history of FVIII inhibitors and increasing inhibitor titer in patients with active inhibitors are thought to be risk factors for increased bleeding risk and morbidity. Furthermore, based on the recent incidence of nAbs formation in the AFFINE trial, it was suggested that the frequency of this adverse event is potentially comparable to inhibitor formation in previously treated patients with HA receiving FVIII prophylaxis [314]. To date, only 2 clinical trials, the GENEr8-INH trial (NCT04684940) and ANB-010-1/EDELWEISS (NCT06185335), have included patients with pre-existing FVIII inhibitors, and preliminary results are available only for Roctavian (phase 1/2 GENEr8-INH). These results are based on four individuals with HA, two with a history of inhibitors (previously tolerated by protein therapy) and two with active inhibitors. The therapy had a similar safety profile as in patients without inhibitors. In the two participants with a prior inhibitor history, there was no inhibitor recurrence, and they achieved therapeutic FVIII levels. In two participants with active inhibitors, an initial anamnestic response occurred. One participant subsequently developed detectable FVIII activity alongside inhibitor decline by week 28, allowing cessation of bleeding prophylaxis. The other maintained a high-titer inhibitor through the data cutoff (week 52) [231,356,357]. The results obtained are ambiguous and raise questions about the economic and medical validity of such studies, given that only 1 out of 2 patients with active inhibitors responded to therapy. However, the duration of the therapeutic effect remains unknown, and long-term follow-up is required. Moreover, current clinical trial criteria exclude anyone who has previously received gene therapy, even if unsuccessfully, making a person ineligible for other AAV-based gene therapies. High cost, uncertain durability, inter-patient variability, impossibility of therapy re-administration, and healthcare system readiness determined the low cost-effectiveness and voluntary withdrawal of Roctavian in 2026 [358].

## 6. Conclusions

This review provides insight into the latest structural, physiological, immunological, preclinical, and clinical studies of factor VIII, as well as its modifications, and the results of AAV-mediated gene therapies for the treatment of hemophilia A.

The development of gene therapy for coagulation disorders is complicated by the difficult synthesis of most hemostatic proteins in heterologous expression systems. Size, correct post-translational modifications, secretion, and controlled activation are critical for different coagulation factors. In this context, FVIII emerges as a particularly challenging engineering candidate. In addition to the great length of the full-length FVIII coding sequence, which exceeds the packaging capacity of AAV, FVIII imposes a significant burden on the ER and the cell’s secretory pathway. FVIII biosynthesis is limited by folding, disulfide bond formation, glycosylation, intracellular transport, and secretion. Therefore, even BDD variants designed to reduce CDS and improve protein yield remain difficult to produce efficiently. The complexity of FVIII engineering is also determined by its inherent function. FVIII is not an enzymatic protein whose catalytic activity can be directly optimized on a purified substrate. Therefore, the functional activity of FVIIIa is determined by the interacting properties of its partner proteins.

A better understanding of the spatiotemporal mechanism of FVIII’s interaction with its partners provides the foundation for improved FVIII variants. The reason for diving into structural updates and our attempt to systematize the accumulated knowledge about FVIII’s molecular partners and structural elements is the observed lack of a clear understanding of the fundamental mechanisms from biosynthesis to FVIII degradation. Given this context, the competition among biotechnology companies to launch drugs onto the market has contributed to the emergence of controversial data (such as regarding the significance of N-glycosylation or the negative role of BiP in secretion) and the prevalence of non-rational design approaches for FVIII.

Recent studies have highlighted the unclear role of the B-domain. On one hand, the B-domain may impair FVIII expression, with its N-glycosylated regions promoting clearance. On the other hand, the B-domain supports folding, prevents aggregation, and contributes to the intracellular transport and secretion of FVIII. FVIII undergoes multistep folding and processing, glycosylation, and receptor-mediated transport in the ER and the Golgi apparatus, with the involvement of the LMAN1-MCFD2 chaperone complex. However, the partial independence of secretion from these factors and the unclear role of the B-domain, glycans, and alternative sorting pathways suggest gaps in current understanding of intracellular processing and protein secretion. Latest structural studies have confirmed that FVIII activation not only releases FVIII from vWF but also converts it into a conformation enabling high-affinity binding to the membrane and assembly of the intrinsic Xase complex. However, the classic model of PS-dependent membrane binding remains incomplete. PS-independent interactions with protein components on the activated platelet surface, including fibrin and αIIbβ3, may provide a significant contribution. Furthermore, recent findings reveal that FIXa stabilizes the A2 subunit of FVIIIa and protects it from APC-dependent inactivation, whereas the role of alternative proteolysis of FVIIIa and glycan-dependent pathways of its clearance remains unclear.

The design of improved synthetic FVIII variants has gradually advanced from direct depletion of the B-domain to point mutations to facilitate secretion. FVIII secretion is enhanced by synthesis in the SC form, preservation of N-glycosylated motifs in the B-domain, attenuation of aggregation in the A1 region, or incorporation of beneficial orthologous substitutions. Hemostatic efficacy is improved by providing protection against APC and increased binding of the complex to FIXa, regardless of enhancing secretion or stabilizing the A2 subunit. Combining multiple mutations appears to have an additive effect in increasing functional activity, which remains a promising strategy. However, it must be considered that some changes improving one stage, such as enhancing secretion, SC assembly, or specific activity, can also decrease stability, alter immunogenicity, or lead to prothrombotic risk.

Early RV and Ad strategies have provided the proof-of-concept for in vivo expression of FVIII and have revealed that liver-specific expression may promote immune tolerance, especially with neonatal transgene delivery. However, RV in vivo gene therapy is complicated by inconsistent FVIII expression in adult animals and the risk of insertional mutagenesis, while Ad in vivo gene therapy is associated with significant immunogenicity and hepatotoxicity, causing loss of expression. These limitations explain the development of less immunogenic vector platforms.

AAV-based therapies are currently the most common treatment for HA based on the number of clinical trials, surpassed only by rFVIII protein-based therapies. The early DC strategy made it possible to overcome the packaging capacity limitations of AAV and create space for stronger, tissue-specific regulatory elements, as well as optimize the ratio of the two chains. However, the efficacy of the DC strategy for hFVIII is limited by a distinct chain imbalance in secretion and the necessary co-expression of both chains within a single cell.

AAV gene therapy design should focus not on maximum expression but on a stable therapeutic activity level with minimal viral load and a decrease in FVIII length. Shortening the FVIII CDS allows for cost reduction in vector manufacturing and enables the use of the most effective regulatory elements, rather than the shortest ones.

The inhibitor eradication following the administration of high doses of AAV8-cFVIII to dogs with HA and the rapid inhibitor formation after the expression of hFVIII in healthy primates highlight the critical role of a combination of various factors, such as transgene specificity, the patient’s immune status, dose, and vector characteristics in the development of tolerance or an immune response [203,259,285,330]. Finally, since clinical data in individuals with active or pre-existing inhibitors remain extremely limited and heterogeneous, the ability of liver-targeted AAV therapy to restore FVIII activity and induce tolerance simultaneously should still be considered a promising, yet unproven, therapeutic strategy. This is especially true given recent reports of neutralizing antibody development following AAV gene therapy in patients with no history of inhibitor formation.

At present, the clinical reliability of AAV-mediated gene therapy in hemophilia A remains debatable. Roctavian, the only FDA-approved HA gene therapy, failed to demonstrate a sustained competitive advantage over prophylactic replacement therapy and was subsequently discontinued. Current limitations include substantial inter-patient variability, rapid loss of FVIII activity, the requirement for the absence of pre-existing anti-AAV antibodies, and the inability to readminister the vector. The investigation and development of novel FVIII variants would improve the efficacy and predictability of gene therapies by enabling stable, long-term therapeutic activity. Gene therapy could represent the most advantageous approach, since endogenous FVIII expression may preserve physiological functions beyond its procoagulant activity, as suggested by recent studies.

## Data Availability

No new data were created or analyzed in this study. Data sharing is not applicable to this article.

## Abbreviations

The following abbreviations are used in this manuscript:

AAT	Alpha-1-antitrypsin
AAV	Adeno-associated virus
ABR	Annualized bleeding rate
Ad	Adenoviral vector
AEs	Adverse events
ALT	Alanine aminotransferase
APC	Activated protein C
aPCC	Activated prothrombin complex concentrate
ASGPR	Asialoglycoprotein receptor
AT	Antithrombin
ATG	Antithymocyte globulin
a.a.	Amino acid
BDD	B-domain deleted
BiP	Binding immunoglobulin protein (GRP78)
cFVIII	Canine factor VIII
CDS	Coding DNA sequence
CHAMP	Centers for disease control and prevention Hemophilia Mutation Projects
CNX	Calnexin
CO	Codon-optimization
CRD	Carbohydrate-recognition domain
Cryo-EM	Cryogenic electron microscopy
CRT	Calreticulin
CSA	Chromogenic assay
DC	Dual-chain
cTFh	Circulating follicular T helper cells
DCs	Dendritic cells
EHL	Extended half-life
ER	Endoplasmic reticulum
ERAD	Endoplasmic-reticulum-associated degradation
ERGIC	ER-Golgi intermediate compartment
EUHASS	European Haemophilia Safety Surveillance
FDA	Food and Drug Administration
FIX (FIXa)	Coagulation factor IX (activated)
FV (FVa)	Coagulation factor V (activated)
FVIII (FVIIIa)	Coagulation factor VIII (activated)
FVIII:Ag	Factor VIII antigen
FVIII:C	Factor VIII procoagulant activity
FVIII-WT	Wild-type factor VIII
FX (FXa)	Coagulation factor X (activated)
HA	Hemophilia A
HB	Hemophilia B
HC	Heavy chain
hFVIII	Human factor VIII
HGVS	Human Genome Variation Society
HMM	Hidden Markov model
HSCs	Hematopoietic stem cells
ITI	Immune tolerance induction
KCs	Kupffer cells
LC	Light chain
LMAN1	Lectin, mannose-binding 1
LRP	Low-density lipoprotein receptor-related protein
LSECs	Liver sinusoidal endothelial cells
LV	Lentiviral vectors
MCFD2	Multiple coagulation factor deficiency protein 2
MMF	Mycophenolate mofetil
mFVIII	Murine factor VIII
MHC	Major histocompatibility complex
MOG	Myelin oligodendrocyte glycoprotein
nAbs	Neutralizing antibodies
NHP	Non-human primate
OSA	One-stage clotting assay
OST	Oligosaccharyltransferase complex
PEG	Polyethylene glycol
pFVIII	Porcine factor VIII
PS	Phosphatidylserine
rFVIII	Recombinant factor VIII
RV	Retroviral vector
SC	Single-chain
SF	Surface fibrin
SHL	Standard half-life
SP	Signal peptide
SPC	Signal peptidase complex
SRP	Signal recognition particle
TAbs	Total antibodies
TFPI	Tissue factor pathway inhibitor
TTR	Transthyretin
UGGT	UDP-glucose:glycoprotein glucosyltransferase
ULVWF	Ultra-large von Willebrand factor strings
UPR	Unfolded protein response
VWF	Von Willebrand factor
WBPs	Weibel–Palade bodies
Xase	Tenase complex
